# Host-Gated Enzymatic Release (H-GER) Enables Colorimetric Transduction for Enzyme Measurement

**DOI:** 10.1021/acsami.5c12410

**Published:** 2025-09-02

**Authors:** Zeyu Zhang, Wen Liu, Qing Huang, Xiang Zhong, Jin Gu, Ruby Segerman, Jordan Choi, Xing Wang, Zhicheng Jin

**Affiliations:** † Department of Chemistry, 1373 Georgia State University, Atlanta, Georgia 30303, United States; ‡ Department of Chemistry, 5452 University of Miami, Coral Gables, Florida 33146, United States; § School of Computing, 7060 The University of Utah, Salt Lake City, Utah 84112, United States

**Keywords:** colorimetric assay, host−guest chemistry, enzyme sensor, cyclic substrate, interfacial kinetics, in vitro diagnostic

## Abstract

We introduce Host-Gated Enzymatic Release (H-GER) as an alternative colorimetric signal transduction mechanism for measuring amylase activity. This assay uses a visually colored complex formed when hydroxypropyl-γ-cyclodextrin (HP-γ-CD) binds to the aggregachromic dye CRANAD-2, with the HP side chains playing a key role in the complexation. The analytical capability of this visually addressable assay relies on changes in dye dispersity, triggered by the enzymatic release of gated CRANAD-2 from HP-γ-CD host. Upon cleavage of HP-γ-CD, the freed dye clusters in the aqueous environment, resulting in a sequence of color changes observed by the naked eye. The H-GER assay demonstrated a limit of detection of 154 U/mL for α-amylase. Analysis based on Michaelis–Menten kinetics and molecular dynamics simulations revealed that the H-GER assay exhibits good enzymatic specificity, despite showing reduced catalytic efficiency. These results demonstrate that H-GER is an effective and potentially valuable signal transduction mechanism that expands the current toolbox for developing *in vitro* colorimetric assays targeting specific enzymatic activities.

## Graphical abstract

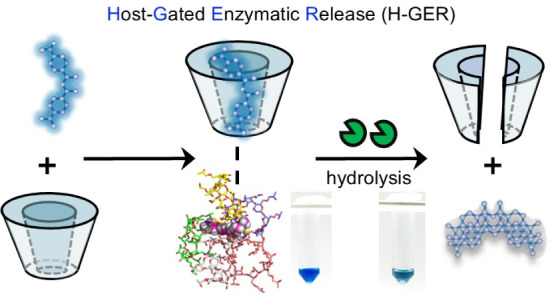

## Introduction

Colorimetric assays for enzyme measurement are indispensable in bioprocessing and *in vitro* diagnostics, offering advantages in cost-effectiveness, portability, speed, and user-friendliness. Their versatility stems from diverse signal transduction mechanisms, including those exploiting controlled colorant dispersity, e.g., gold nanoparticle aggregation assay, forensic Phadebas test, health PrettyLitter technique.^1–9^ Indicator displacement assays (IDAs) exemplify the colorant dispersity principle through competitive host–guest binding, in which target analytes displace immobilized reporters from host cavities, reporting optical signals through color shifts.^10,11^ Despite their exceptional adaptability stemming from a vast library of cyclic host molecules, conventional IDAs are poorly suited for macromolecules like proteins and enzymes, highlighting the need for alternative mechanisms to overcome this limitation.

We introduce host-gated enzymatic release (H-GER) as an alternative to IDAs for enzyme measurement, where the target enzyme selectively cleaves a macrocyclic host, releasing gated colorants whose dispersity changes produce visible color signals. The key distinction between H-GER and established colorimetric methods, such as nanoparticle aggregation assays, is that most nanoparticles operate merely as colorants without any defined interactions with the recognition substrate and can even be added exogenously, whereas H-GER requires colorants to be specifically complexed with an enzyme-degradable substrate, making it necessary to screen for optimal host–colorant complexes, as in IDAs.^3,12^ Enzyme-degradable cyclic hosts–including cyclodextrins (CDs), cyclopeptides, cyclooligosaccharides, and cyclophosphates–offer tunable cavities with programmable binding affinity, selectivity, biocompatibility, and synthetic versatility.^13^ Additional cyclic substrates, such as esterase-sensitive polyesters and oxidase-labile boronate cages, also serve as viable hosts for H-GER systems.^14–16^ Among, CDs are attractive cyclic hosts due to their low cost,^17,18^ high biocompatibility, and diverse structures–including tunable cavity sizes and amphiphilicity. The α-1,4-glycosidic linkages in cyclodextrins are inherently susceptible to salivary and pancreatic α-amylase, illustrating how substrate recognition and enzymatic selectivity can be encoded into the host’s molecular topology.^19^ Therefore, we hypothesize that CD-based H-GER systems will yield color signals correlate to α-amylase activity. Such rational assay design remains challenging due to the complex and elusive nature of the complexed substrate–dye interface and its influence on enzyme–substrate interactions.^20–22^

To test our hypothesis, we screened eight dextrin derivatives and four dye reporters, identifying hydroxypropyl-γ-CD (HP-γ-CD) and CRANAD-2 (an aggregachromic dye) as a proof-of-concept H-GER system for α-amylase measurement. UV–Vis spectroscopy revealed that amylase-mediated degradation of CRANAD-2⊂HP-γ-CD led to a decrease in the main absorbance peak and the emergence of 441 nm band, indicating the release and subsequent aggregation of CRANAD-2. The stoichiometric ratio of HP-γ-CD to CRANAD-2 was determined to be 200:1, which forms stable complexes. Optical titration experiments determine the disassociate constant to be *K*_d_ = 25.14 mM. Proton nuclear magnetic resonance (^1^H NMR) and dynamic light scattering (DLS) analyses confirm successful complexation. Molecular dynamics (MD) simulations indicate the gating mechanism by showing that the hydroxypropyl side chains’ oxygen atoms promote hydrogen bonding with the boron–fluorine in CRANAD-2, where five HP-γ-CD stabilizes one dye rather than forming an equal molar inclusion through cavity penetration. We also found that BSA block is the key for stabilizing the H-GER system during subsequent α-amylase measurement. The limit of detection (LoD) for α-amylase was estimated to be 154 U/mL, and kinetics analysis yielded the specificity constant to be *k*_cat_/*K*_m_ = 1.02 M^–1^ s^–1^. This specificity constant is 3 orders of magnitude lower than that of free CDs by α-amylase digestion, likely due to competitive substrate–dye interactions, the observed inhibition at excess substrate concentrations, and restricted amylase accessibility to substrates in the complexes. Our rational H-GER design is adaptable to target other enzymes, including hydrolases, proteases, and oxidoreductases,^23,24^ thereby expanding the mechanistic toolbox for *in vitro* colorimetric assays.

## Experimental Section

### CRANAD-2 Synthesis

Acetylacetone (10 mmol, 1.03 mL) was dissolved in anhydrous dichloromethane (50 mL) under a nitrogen atmosphere. Boron trifluoride diethyl etherate (15 mmol, 1.85 mL, 1.5 equiv) was added dropwise to the solution over 5 min at room temperature. The reaction mixture was then heated to reflux (40 °C) and stirred overnight. After cooling to room temperature, the reaction was quenched by the addition of water (15 mL) and stirred for an additional 10 min. The organic layer was separated, and the aqueous phase was extracted with dichloromethane (3 × 15 mL). The combined organic layers were washed with water, dried over anhydrous Na_2_SO_4_, and filtered. The filtrate was concentrated under reduced pressure to afford the crude product, which was used directly in the next step without further purification. ^1^H NMR (400 MHz, DMSO-*d*_6_) δ 6.41 (s, 1H), 2.36 (s, 3H), 2.36 (s, 3H).

A drop of piperidine was added to a solution of the 4-dimethylaminobenzaldehyde (4.0 mmol) and 2,2-difluoro-1,3-dioxaboryl-pentadione (0.8 mmol) in dry acetonitrile (10 mL). The reaction mixture was stirred under reflux for 2 h. The reaction was then quenched with water (50 mL), and the aqueous phase was extracted with dichloromethane (3 × 50 mL). The combined organic layers were dried over anhydrous Na_2_SO_4_, filtered, and concentrated under reduced pressure. The crude product was purified by silica gel column (5 × 50 cm) chromatography to afford CRANAD-2. ^1^H NMR (400 MHz, DMSO-*d*_6_) δ 11.01 (d, *J* = 15.4 Hz, 2H), 10.86 (d, *J* = 8.6 Hz, 4H), 9.97 (d, *J* = 10.6 Hz, 6H), 9.47 (s, 1H), 6.25 (s, 12H).

### Preparation of CRANAD-2⊂HP-γ-CD Complex

A stock solution of CRANAD-2 (3.66 mM) was prepared in DMSO. The molar extinction coefficient of CRANAD-2 at its absorption maximum is 1.34 × 10^5^ M^–1^ cm^–1^ in DMSO. To make CRANAD-2⊂HP-γ-CD, 16.4 μL of the CRANAD-2 solution (stock sample, 60 nmol) was transferred to a microcentrifuge tube, followed by the addition of 400 μL of HP-γ-CD in DMSO (30 mM, 3 μmol) to achieve a 200:1 molar ratio (HP-γ-CD-to-CRANAD-2). The resulting mixture was sonicated for 40 min and subsequently stirred magnetically at 37 °C overnight in darkness. The solvent was then removed using a vacuum concentrator (Vacufuge, Eppendorf) operated at 30 °C. For subsequent experiments, the dried complex was reconstituted in 400 μL of deionized water or desired media, yielding a deep blue solution.

### MD Simulations

MD simulations were conducted using GROMACS 2024.51 program. Two systems, (1) bound: CRANAD-2 and HP-γ-CD; (2) unbound: CRANAD-2 and linear disaccharide (refer to as CD unit); were prepared in the cubic box by packmol software with the length 16 nm. To ensure consistent initial configurations and minimize artificial clustering, the CRANAD-2 molecules were spatially evenly distributed and positioned in both systems. The OPLS-AA force fields were employed to model bonded and nonbonded interactions. The 1.14*CM1A-LBCC were used to assign atomic partial charges. Nonbonded interactions were excluded in less than three bonds, and standard 1–4 pair interactions were explicitly reintroduced, with LJ interactions scaled by 0.5 and Coulombic interactions scaled by 0.8333 for those pairs, consistent with OPLS-AA parametrization. The Verlet cutoff scheme was employed for neighbor searching, with a 1.6 nm cutoff applied to both van der Waals and electrostatic interactions. All simulations were performed under periodic boundary conditions in three dimensions. Long-range electrostatic interactions were calculated using the Particle Mesh Ewald method, with a fourth-order interpolation scheme, following long-range energy and pressure corrections. LINCS algorithm were applied to constrain hydrogen bonds with an expansion order of four and a maximum constraint angle of 30 degrees to maintain numerical stability during integration. The initial structures were energy minimized using the steepest descent algorithm with a maximum force convergence threshold 100 kJ/mol/nm. After minimization, the systems were equilibrated in two stages: (a) NVT ensemble (constant volume and temperature): The system was equilibrated for 1 ns at 310.15 K using the velocity-rescale thermostat with a time constant of 5 ps. (b) NPT ensemble (constant pressure and temperature): A subsequent 1 ns equilibration was performed under isotropic pressure coupling at 1 bar using the Parrinello–Rahman barostat with the same thermostat settings as in the NVT stage. Following equilibration, the final production MD simulations were carried out for 200 ns under the same NPT conditions. During this phase, coordinates were saved every 10 ps; energy terms were recorded every 4 ps; center-of-mass motion was removed every 2 ps. This protocol enabled robust sampling of dye molecule dynamics while maintaining thermodynamic stability and physical consistency throughout the trajectory.

### LoD Measurement

To evaluate the enzyme-responsiveness of the CRANAD-2⊂HP-γ-CD complex, dried samples (150 μM of CRANAD-2) prepared at a 200:1 molar ratio (CD:dye) were reconstituted in 400 μL of deionized water. Different doses of α-amylase were added during reconstitution to achieve final concentrations of 10, 60, 120, 500, 1200, 2000, 4000, 6000, 9000, 12,000 U/mL, enabling an enzyme concentration-dependent response study. The resulting solutions were immediately transferred into wells of a 96-well microplate preblocked with 1% BSA. The plate was sealed and placed into plate reader (Varioskan LUX, Thermo Scientific) set to 37 °C. Absorbance spectra were continuously recorded for 16 h. Each condition was measured in triplicate (*n* = 3), and absorbance values at 594 nm and 441 nm were extracted for ratiometric analysis (Abs_594/441 nm_) to monitor enzymatic degradation of the complexes over time. Details of the LoD calculation are provided in Section S6.2.

### Enzyme Kinetic Assays

To evaluate the enzymatic kinetics of the CRANAD-2⊂HP-γ-CD system, a series of reactions were performed using increasing substrate concentrations under a fixed α-amylase level (9960 U/mL). The CRANAD-2⊂HP-γ-CD complexes at 200:1 stoichiometry were weighed and reconstituted in deionized water to obtain final substrate concentrations ranging from 1.5, 3, 6, 12, 18, 24, 30 μM (refer to as CRANAD-2 concentration). α-Amylase was then added to each sample to reach a final concentration of 9960 U/mL. The total reaction volume was 400 μL. Following gentle vortexing, each sample was transferred into a 96-well flat-bottom microplate preblocked with 1% BSA. The microplate was sealed and incubated at 37 °C inside a plate reader. Absorbance spectra were continuously recorded for 16 h within the 350–800 nm range. Ratiometric analysis (Abs_594/441 nm_) was used as the optical readout for substrate degradation. Reaction velocities were calculated based on the initial rate of signal change within the experimentally defined linear window. All measurements were performed in triplicate (*n* = 3), and resulting data were used to construct a Michaelis–Menten plot and fit for kinetic parameters (e.g., *k*_cat_ = *V*_max_/[E] with α-amylase to be 320 U/mg and 55 kDa; *K*_m_ is defined as the Michaelis constant, which represents the substrate concentration at which the enzyme reaction rate is half of its maximum velocity, *V*_max_).

## Results and Discussion

### Rationale

Validating the H-GER system as an alternative colorimetric transduction mechanism was motivated by the need to expand IDAs for detecting biomacromolecules, particularly enzymes. Over the past four years, we have developed nanoparticle-based colorimetric systems which, although effective and intense, require complex surface functionalization.^3,12,22,25,26^ In contrast, biocompatible chromophores remain the most widely used colorants in commercial biosensors, as highlighted in our recent review.^3^ Among these, CRANAD-2—a dye originally developed for in vivo imaging of amyloid-β aggregates—prompted our exploration of how cyclic molecules influence its dispersity and colors in aqueous media.^27^ Many other color changes with dye dispersity modulation have been reported, underscoring the potential of this approach to diversify current colorimetric strategies.^27,28^

In this study, we selected α-amylase (a Ca^2+^-dependent glycosidase) as a model enzyme due to its relevance in industrial beverage processing, oral health monitoring (e.g., in electronic cigarette users and diabetic patients),^29,30^ and forensic analysis.^1,31^ We will refer to α-amylase as amylase for the remainder of the study. We then hypothesize that enzymatic cleavage of the cyclosubstrate releases the encapsulated aggregachromic dyes, triggering dye clustering and color signals (Figure 1). The H-GER system was optimized using four dyes and eight dextrin derivatives. Complex formation was characterized by optical spectroscopy, ^1^H NMR, DLS, and MD simulations. Analytical performance was evaluated based on operation concentrations, time windows, LoD, kinetic analysis, specificity test, and matrix effects.

**Figure 1:**
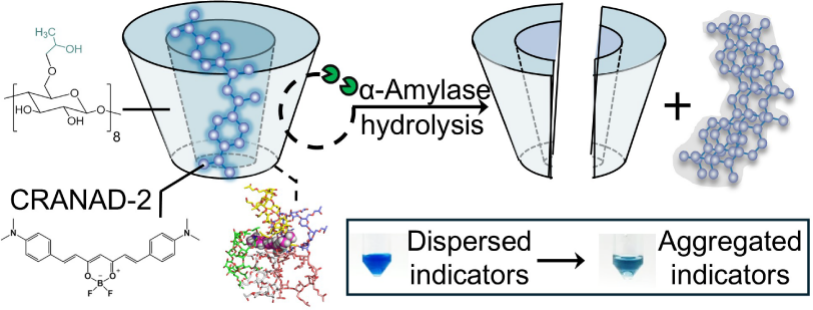
Schematic illustration of the H-GER colorimetric mechanism using the CRANAD-2⊂HP-γ-CD complex and amylase. The simulated stoichiometry for HP-γ-CD:CRANAD-2 is 5:1. Enzymatic hydrolysis of the complexes by α-amylase releases the dispersed CRANAD-2, leading to dye aggregation and a visible color shift from initial blue to intermediate cyan.

### Synthesis and Characterization of H-GER Complexes

We simplified the screening into two steps: (i) identifying the optimal dextrin variant for maximizing dye dispersibility in aqueous solution, and (ii) selecting aggregachromic dyes that exhibit both absorbance intensity and wavelength shifts in the H-GER systems. As shown in Figure 2a,b, the tested materials include eight dextrin variants [i.e., α-CD, β-CD, γ-CD, their hydroxypropylated derivatives, linear trisaccharide (LT), and glucose monomer (GM)] and four dyes (i.e., methylene blue, curcumin, Nile Red, and CRANAD-2). Experimentally, we identified three key conditions for successful synthesis of H-GER complexes: (i) pure dimethyl sulfoxide (DMSO) solvent ensures a full dissolution for efficient complexation; (ii) solvent removal by vacuum centrifugation produces a dense pellet and strong color upon redispersion, likely due to enhanced molecular packing, whereas freeze-drying yields pale colors (Figure S2a); (iii) a minimum CD-to-dye molar ratio of approximately 200:1 is required for a full color restoration in aqueous media; and (iv) the microtubes for rehydration must be preblocked with 1% w/v bovine serum albumin (BSA) to minimize nonspecific adsorption of H-GER complexes to polypropylene surfaces (Figure S2b). The resulting H-GER complexes restore the coloration that resembles monodispersed dye in aqueous solution. We attribute this to the dye remaining dispersed due to its immobilization and complexation with cyclodextrins.

**Figure 2:**
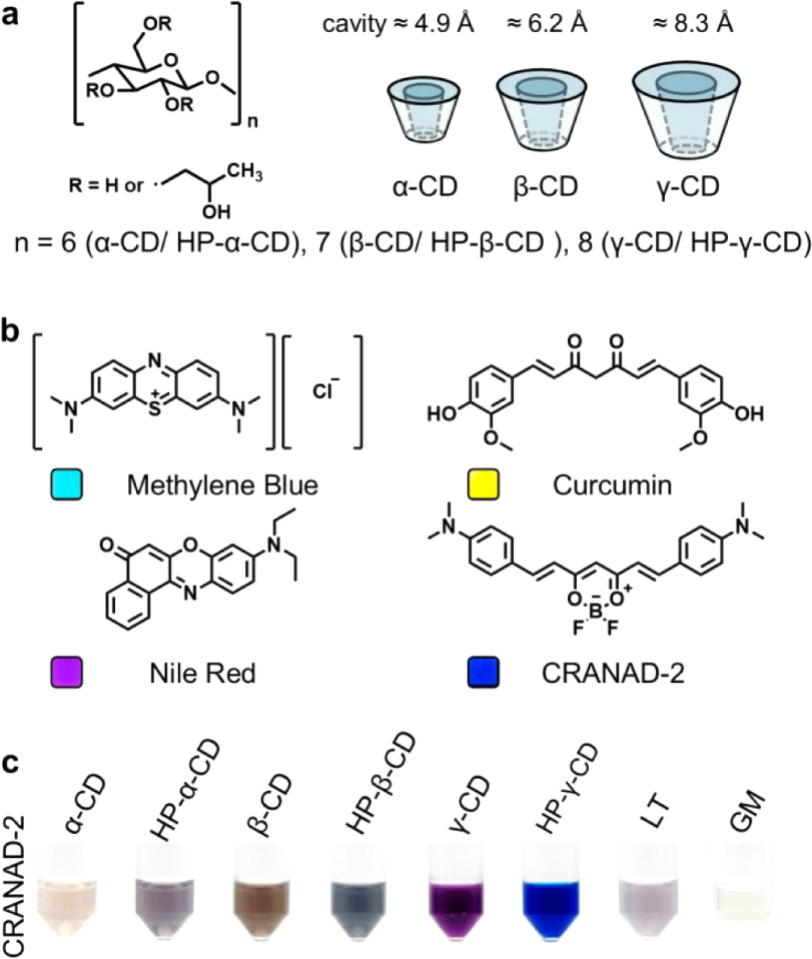
Material optimization of the H-GER system. (a) Chemical structure and size of native and hydroxypropyl (HP)-modified cyclodextrins including α-, β-, and γ-CD. (b) Chemical structure of four colorants used, including methylene blue (cyan), curcumin (yellow), Nile Red (purple), and CRANAD-2 (blue). (c) White-light photo of CRANAD-2 complexed with various native and modified dextrins, as well as linear trisaccharide (LT) and glucose monomer (GM) controls. HP-γ-CD fully restores blue color of CRANAD-2 in aqueous media.

#### Optimal Dextrin Variant

To screen the best dextrin variant, we selected CRANAD-2 due to its remark color sensitivity to polarity (e.g., blue in DMSO, purple in ethyl acetate; Figure S3a) and,^32^ as confirmed in this study, its versatile color shifts in aqueous media. CRANAD-2 contains a central BF_2_-chelated diketone for hydrogen bonding and hydrophobic π-conjugated arms for van der Waals (vdW) interactions, making it a potential guest molecule for cyclodextrin complexation.^28^ The toroidal cavity of HP-γ-CD provides a noncovalent environment that offers both physical confinement and partial shielding of hydrophobic guests. Among the eight tested dextrin variants (molar ratio = 200:1, dextrin:dye), only HP-γ-CD produced intense coloration upon rehydration (Figure 2c), indicating successful H-GER complexation. Notably, HP-γ-CD fully restored the characteristic deep blue color of dispersed CRANAD-2, which is otherwise unattainable in water. In contrast, α-CD, β-CD, and their hydroxypropylated forms yielded colorless or turbid suspensions, likely due to low degree of hydroxypropylation and insufficient cavity size (4.9–6.2 Å vs 8.3 Å for γ-CD) to accommodate CRANAD-2 (18 × 11 × 4 Å^3^).^27,33^ We note that host suitability is dye-specific, e.g., curcumin and Nile Red are best encapsulated by HP-β-CD and β-CD, as reported previously.^34–37^

The specificity of CRANAD-2⊂HP-γ-CD interactions was further confirmed using two noncyclic saccharide controls—a linear trisaccharide and a glucose monomer—which produced purple gray or no perceptible color under identical conditions (Figure 2c), demonstrating the necessity of the macrocyclic structure for H-GER complexation. Oligosaccharides are the conversion of certain cyclodextrins by α-amylase.^38,39^ These critical controls support our hypothesis that enzymatic digestion of cyclodextrin complexes would potentially induce color changes.

#### Aggregachromic Dyes

We next investigated whether HP-γ-CD could encapsulate across other aggregachromic dyes—methylene blue, Nile Red, and curcumin—and whether tuning the CD-to-dye molar ratio (0 to 10,000) modulates color transformation.^40^ Here, we adopt the term “aggregachromic” as describing a chromophore whose optical properties change upon dispersion or aggregation.^41^ As shown in Figure 3a, methylene blue retained its native color and absorbance regardless of HP-γ-CD concentration (Figure S3), as its hydrophilic nature eliminates the need for complexation to maintain coloration. In comparison, Nile Red and curcumin intensify color with increasing HP-γ-CD concentrations. Nonetheless, neither dye showed color transformation nor spectral shifts (Figure S3).

**Figure 3:**
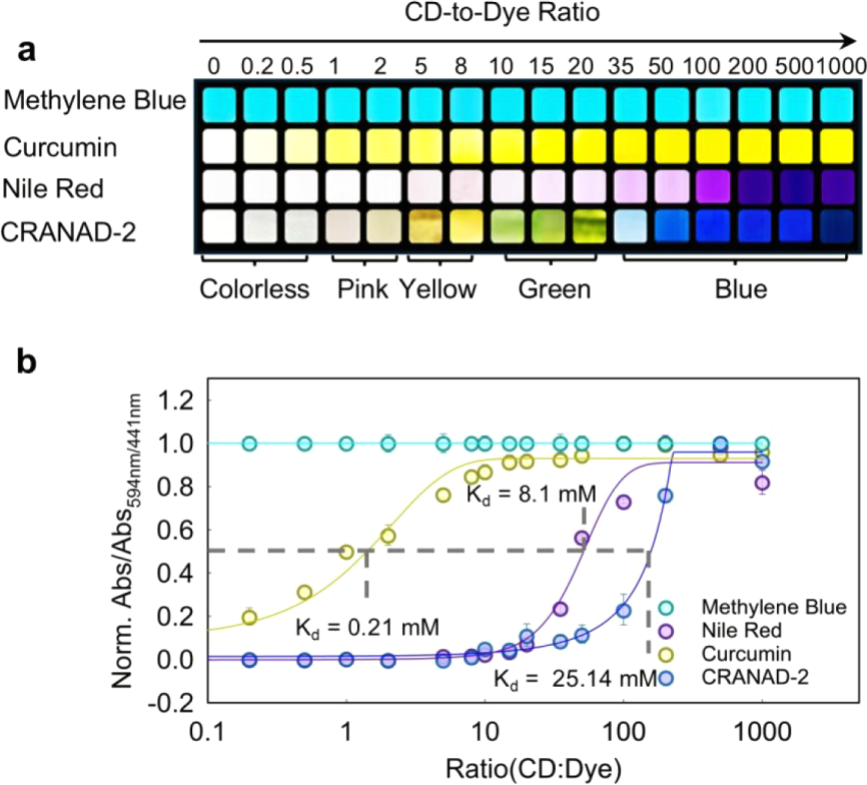
Optimization of CD-to-dye ratio in H-GER systems. (a) Photographic illustration of color transformation in four dyes—Methylene Blue, Curcumin, Nile Red, and CRANAD-2—upon titration with increasing molar ratios of HP-γ-CD from 0 to 1,000. Note that photos were taken at 10 min after complex rehydration. CRANAD-2 exhibits a versatile color transition from colorless to yellow, green, and blue. (b) Normalized absorbance response of each dye as a function of the CD-to-dye ratio. For CRANAD-2, ratiometric Abs_591/441 nm_ was employed, while for the other dyes, the intensity at their respective maximum absorbance wavelengths was used. Apparent binding was fitted into sigmoidal curves. CRANAD-2 showed a slow transition with *K*_d_ = 25.14 mM (or at 200:1 CD-to-dye ratio), whereas Curcumin and Nile Red showed *K*_d_ = 0.21 mM and 8.1 mM, respectively. Methylene Blue showed minimal spectral change and thus no complexation.

In comparison, CRANAD-2 exhibited a CD concentration-dependent, three-stage color transformation—from the initial blue, to intermediate green, pink, and yellow, and finally to colorless—as the CD-to-dye molar ratio decreased (Figure 3a). These visible changes correlated with a progressive shift in the absorbance profile: a decrease at 594 nm and a concomitant increase at 441 nm (Figure S3d), indicating molecular reorganization or electronic modulation.^32^ This 47 nm blue shift in absorbance aligns well with previous reports from Ran’s group, which demonstrated that CRANAD-2 undergoes a 90 nm blue shift upon interaction with amyloid-β aggregates.^27^ Experimentally, ratios above 5:1 produced an immediate blue color upon rehydration, but the color was unstable (Figure S9). A deep blue coloration was then obtained at an HP-γ-CD-to-CRANAD-2 ratio of at least 200:1, with complexes at or above this stoichiometry remaining stable for at least 12 h in BSA-blocked microtubes (Figure S2b). This formulation was therefore adopted for subsequent experiments.

We also analyzed the spectroscopic responses of dye⊂CD complexes across varying host-to-guest molar ratios. For CRANAD-2, we monitored the ratiometric absorbance Abs_594/441 nm_, while for curcumin and Nile Red, we tracked their respective absorption peaks, λ_max_. As shown in Figure 3b, the absorbance of three dyes exhibited sigmoidal curves with increasing HP-γ-CD concentration. Using the concept of dissociation constants (*K*_d_)—defined as the CD concentration required for half-maximal dye binding—we determined stoichiometries of 1 CD for curcumin, 50 CDs for Nile Red, and 200 CDs for CRANAD-2. These stoichiometries differ significantly despite that curcumin, Nile Red, and CRANAD-2 share similar size and solubility (i.e., log *p* = 3.0–3.5).^27,42^ The 1:1 CD-to-curcumin ratio aligns with prior reports (2:1 to 1:2 for other CD variants),^34,35^ while Nile Red and CRANAD-2 require 50- and 200-fold higher HP-γ-CD concentrations, respectively. This disparity likely stems from their increased rigidity and planarity, which limit efficient inclusion within the HP-γ-CD cavity and necessitate high host concentrations to favor complexation by mass action.^43^ This observation is further supported by the MD simulations described below. In comparison, the control dye, methylene blue, exhibited a constant absorbance value at its λ_max_ regardless of host concentration.

### Other Characterizations

#### NMR Spectroscopy

NMR spectroscopy is highly sensitive to intermolecular interactions, as changes in chemical shift and peak shape reflect the local electronic environment.^44^ To probe the formation of CRANAD-2⊂HP-γ-CD complexes, we compared their ^1^H NMR spectra in D_2_O, a complex-promoting solvent, and DMSO-*d*_6_, a complex-disrupting solvent. As shown in Figure 4a,b, the spectrum in D_2_O exhibits broadened and attenuated resonances between 6–8 ppm. In contrast, sharp and well-resolved peaks are observed for the dye reporters in DMSO-*d*_6_, reflecting free CRANAD-2 molecules interacting preferentially with the solvent. The observed line broadening in D_2_O is consistent with restricted molecular motion and slower transverse relaxation, commonly associated with supramolecular encapsulation or complexation.^45–47^ In addition, the observed 0.4–0.8 ppm upfield shift of CRANAD-2 in D_2_O compared to DMSO-*d*_6_ can be attributed to solvent effects. This shift is also indicative of a nonpolar environment, consistent with CRANAD-2 being centered within the hydrophobic, electronically shielding HP-γ-CD molecules. Attempts to further characterize the complexation using two-dimensional NMR techniques proved challenging due to the high CD concentration (200-fold molar excess) and reduced signal intensity of CRANAD-2 protons due to slow transverse relaxation within the complexes.^48^

**Figure 4:**
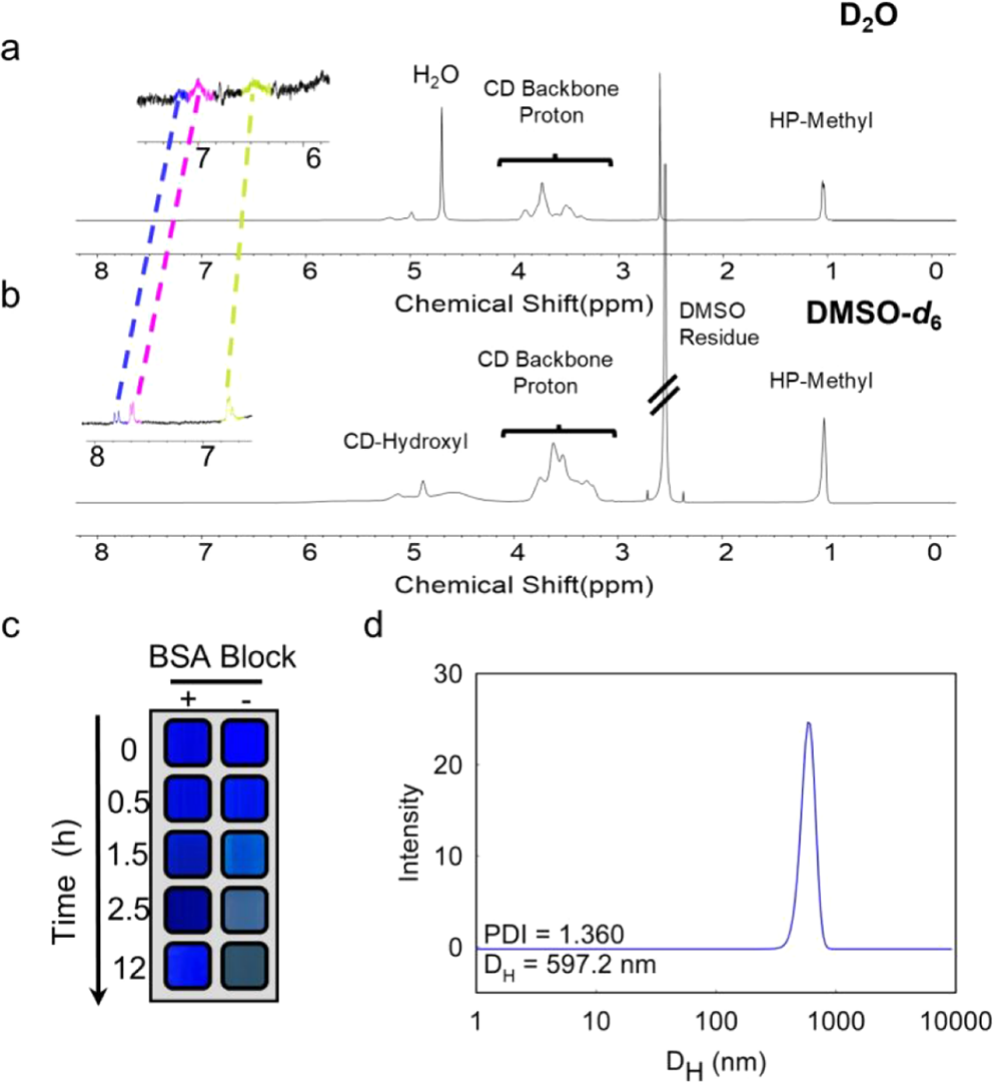
NMR and DLS characterization. ^1^H NMR spectra of the CRANAD-2⊂HP-γ-CD complex recorded in D_2_O (a) and DMSO-*d*_6_ (b). Characteristic broadening in the aromatic proton region (δ 7–8 ppm, color highlighted) in complex-promoting D_2_O confirms the complexation of CRANAD-2 with CDs. (c) Colors of rehydrated CRANAD-2⊂HP-γ-CD with (+) and without (−) BSA blocking over 0–12 h. Without BSA, color fades after 1.5 h due to nonspecific adsorption, while BSA blocking preserves the signal for 12 h. (d) DLS analysis of the freshly prepared CRANAD-2⊂HP-γ-CD, showing the cluster-like complexes with hydrodynamic diameter (*D*_H_) at 597.2 nm and polydispersity index (PDI) > 1.

#### Stability Characterization

To characterize the stability of CRANAD-2⊂HP-γ-CD complexes in aqueous environments, we monitored their color retention in microtubes at 37 °C for over 12 h. We found that BSA blocking on the microtubes is key to stability. As shown in Figure 4c, the BSA-blocked group maintained a consistent deep blue color throughout the entire time period, indicating high colloidal stability. In contrast, the unblocked group exhibited progressive signal loss, with a visible color fading at 1.5 h. BSA likely coats the polypropylene surface, preventing competitive interactions between CRANAD-2, the tube, and cyclodextrin. This highlights the importance of surface passivation in minimizing nonspecific adsorption and false positives—common issues in colorimetric assays.^49,50^ These findings establish BSA blocking as a simple and effective strategy to stabilize the H-GER system, especially in polypropylene microtubes.

#### DLS Measurement

The formation of CRANAD-2⊂HP-γ-CD complexes at 200:1 host-to-guest ratio may reflect either (i) dynamic binding, where CRANAD-2 transiently hops among many free CDs,^51^ or (ii) supramolecular clustering, where a single CRANAD-2 is surrounded by multiple CD molecules.^52,53^ DLS measurements reveal a hydrodynamic diameter (*D*_H_) of 500 nm (polydispersity index, PDI > 1), indicating a broad size distribution and supporting the latter scenario of cluster-like assemblies (Figure 4d). In comparison, the *D*_H_ of pure HP-γ-CD is not detectable by DLS. Thus, the structural heterogeneity of CRANAD-2⊂HP-γ-CD complexes is critical and informative, as it may impair interfacial enzyme kinetics and thus sensing performance due to reduced enzyme–substrate encounter frequency and restricted substrate orientation, as discussed in our previous studies.^22,54^

### Simulations of the H-GER Complexes

Next, we used MD simulations to investigate system’s complex dynamics, intermolecular interactions, and binding modes. Two systems were defined: (i) a “Bound” system with 10 CRANAD-2 molecules and 200 HP-γ-CD molecules, and (ii) an “Unbound” system with 10 CRANAD-2 molecules and 800 noncyclic disaccharides representing α-amylase-cleaved HP-γ-CD (Figure S5a,b). To ensure consistent initial configurations and minimize artificial clustering, CRANAD-2 molecules were evenly distributed in both systems, with an average pairwise distance of 4–5 nm (Figure 5a).

**Figure 5:**
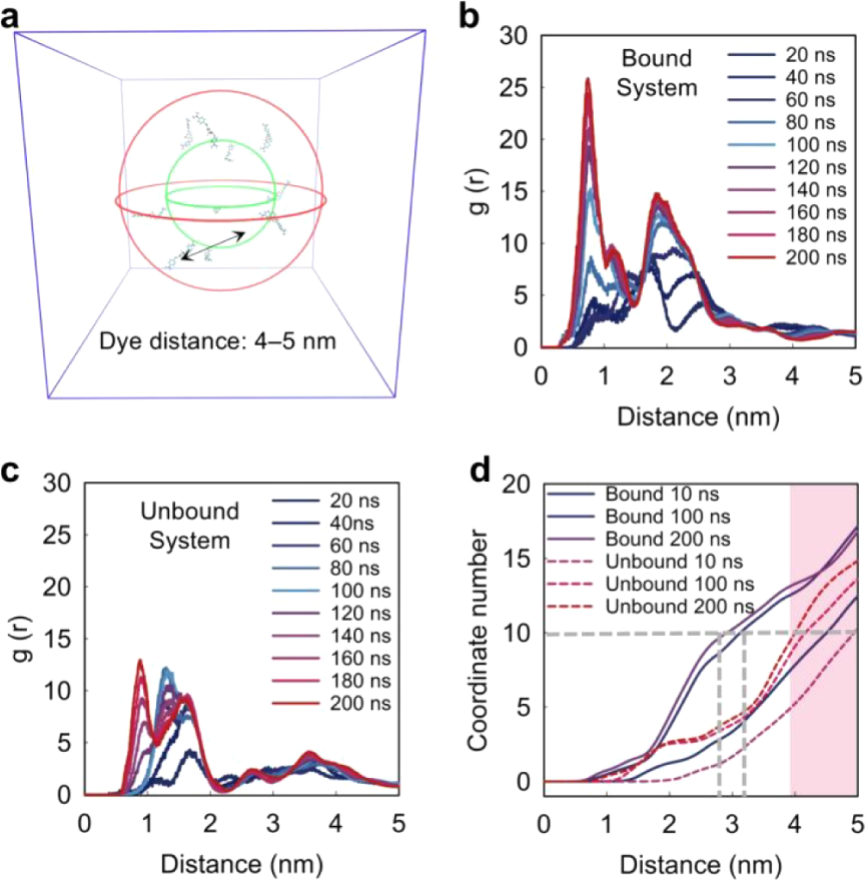
Simulation of system complexity by RDFs. (a) Ten molecules of CRANAD-2 were evenly distributed in the simulated systems, with an average pairwise distance of 4–5 nm. RDFs between dye molecules for the Bound system (b) and Unbound system (c) as a function of time during the MD simulations. (d) In the unbound system, the initial coordination of 10 CRANAD-2 molecules with pairwise distances of 4–5 nm was maintained after 200 ns. In contrast, the bound system showed a reduction in pairwise distances to around 3 nm, indicating increased system complexity due to CRANAD-2⊂HP-γ-CD complexation.

#### System Dynamics and Complexity

After 200 ns of MD simulation, the potential energy profiles of the Bound and Unbound systems indicated that equilibrium was reached (Figure S5c). Subsequent analyses were performed using these equilibrated trajectories. We applied radial distribution functions (RDFs) to analyze the behavior and organization of molecular systems over time. In this study, the integrated RDFs, *g*(*r*), for two systems were computed by TRAVIS program^55^ over 200 ns MD simulation, sampled at 10 ns intervals.

In Figure 5b,c, the *x*-axis represents dye molecules pairwise distances in nanometers, and the *y*-axis denotes the integrated RDFs, offering a cumulative measure of neighboring particles within a given radius. Specifically, the RDFs of the Bound system exhibit a sharp first peak around 0.8 nm and a well-defined second peak near 1.8 nm (Figure 5b), with curves plateauing after 3 nm, especially beyond 100 ns. In comparison, the Unbound system shows a broader and less intense initial peak at 0.9 nm, indicating a loose local structure (Figure 5c). Subsequent peaks are poorly defined, and the RDFs continue rising beyond 2.5 nm without reaching a clear plateau even at 3.5 nm. These patterns suggest that the Bound system forms multiple types of clusters or multishell arrangements with varying characteristic distances, reflecting a more intricate, complex, and dynamic molecular organization. The Unbound system shows uniform RDFs with minimal temporal variation and an evenly dispersed structure, indicating that the initial dye organization remains largely unchanged due to weak interactions following the introduction of noncyclic disaccharides. The preserved distribution of CRANAD-2 in the Unbound system is further supported by coordination number analysis, which shows stable interdye distances using a cutoff that includes all ten dye molecules (Figure 5d, dashed line). In contrast, the Bound system exhibits a reduction in pairwise distances from the initial 4–5 to 3 nm, indicating increased system complexity due to CRANAD-2⊂HP-γ-CD complexation.

#### Interaction Modes in Complexes

We used MD simulations to investigate the interaction modes between CRANAD-2 and HP-γ-CD. To monitor equilibration, we calculated the radius of gyration (Rg) over time (Figure 6a). Both systems started from the same initial dye configuration and showed increasing Rg values, reflecting structural relaxation from the constrained starting state. After 178 ns, the Rg curves stabilized, indicating equilibration—around 7.5 nm for the Bound system and 6.5 nm for the Unbound. The Bound system displayed larger, noisier fluctuations during the system evolution, likely due to transient conformational changes and more complex dynamics. In contrast, the Unbound system remained smoother and more stable, consistent with retention of its initial dispersed structure, aligning well with the above RDF analysis.

**Figure 6:**
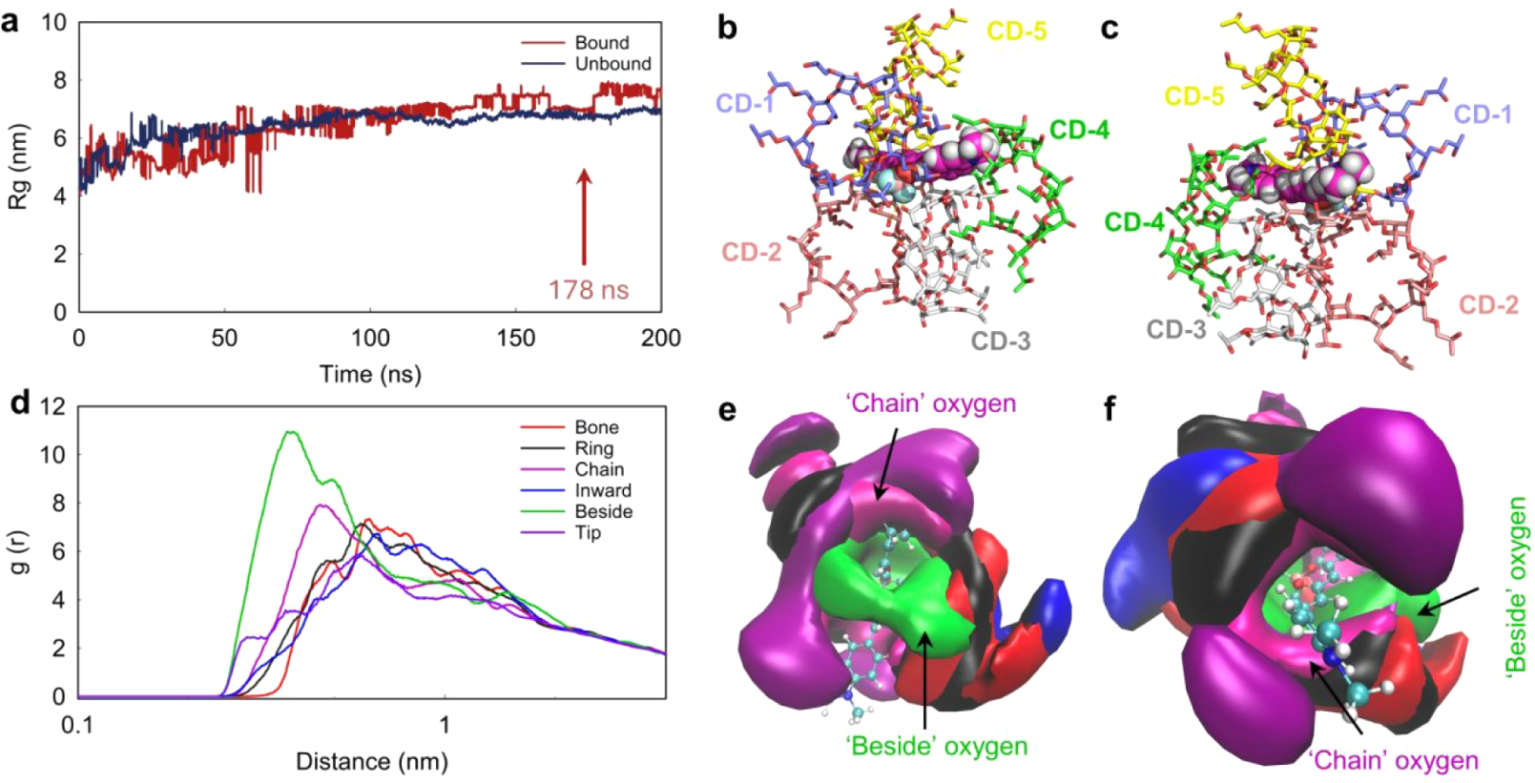
Simulation of interaction modes in the CRANAD-2⊂HP-γ-CD complexes. (**a**) Radius of gyration (Rg) plateaued for both Bound and Unbound systems at 178 ns, indicating structural relaxation and system stabilization. Subsequent analyses were performed using this equilibrated state. (**b**,**c**) Representative cluster views showing one CRANAD-2 molecule (magenta spheres) interacting with five HP-γ-CD molecules (colored sticks), corresponding to a simulated 5:1 CD-to-dye stoichiometry. (**d**) Spatial distribution functions (SDFs) of oxygen atoms from various HP-γ-CD moieties surrounding CRANAD-2, with position and color codes as follows: beside (green), bone (red), chain (magenta), inward (blue), ring (black), and tip (purple). See Figure S5a for color-coded oxygen positions in CD structure. (**e**, **f**) 3D spatial density maps illustrating the distribution of oxygen atoms from distinct structural positions within HP-γ-CD around CRANAD-2.

We then performed cluster analysis using the GROMOS method (g_cluster command),^56^ and MDAnalysis with a 5 Å cutoff.^57^ The representative structure view at 178 ns for the Bound system was extracted for further analysis (Figure 6b,c). In this stabilized cluster, the simulated HP-γ-CD-to-CRANAD-2 stoichiometry was 5:1. CRANAD-2 (magenta spheres) served as the reference center, surrounded by five HP-γ-CD molecules color coded in blue (CD1), red (CD2), gray (CD3), green (CD4), and yellow (CD5). Although chemically identical, these CDs adopt distinct spatial orientations relative to a CRANAD-2, indicating diverse binding modes. The CD2 and CD3 form a cradle-like structure, likely serving as primary interaction partners, while the CD4 and CD1 contribute to peripheral stabilization. The consistent spatial pattern observed across the dominant cluster suggests a thermodynamically favored gating configuration, stabilized by specific hydrogen bonding and hydrophobic contacts. These simulated findings align with our observations that a 5:1 ratio of HP-γ-CD to CRANAD-2 can instantly restore the complex to a blue color upon rehydration, although a 200-fold molar excess is needed for stability. In addition, the simulated arrangement, in which excess host molecules encircle the central dye rather than occupying a conventional 1:1 cavity, is also consistent with the experimentally determined weak binding affinity (*K*_d_ = 25.14 mM, Figure 3b). These simulation results highlight the value of MD simulation for rational H-GER system design and optimization.

We further analyzed the RDFs of the complexes by focusing on the oxygen, boron, and fluorine atoms of CRANAD-2 and various types of oxygen atoms in HP-γ-CD, as color-/position-coded in Figure S5a. Figure 6d shows that oxygen atoms in the “beside” hydroxyl and along the hydroxypropyl “chain” of the CD exhibit the strongest interactions with CRANAD-2. This finding is supported by the spatial distribution functions (SDFs) in Figure 6e,f. The SDF is a statistical method that characterizes the three-dimensional spatial distribution of specific atoms or molecules—here, HP-γ-CD—around a reference group, CRANAD-2. The results reveal that CRANAD-2 is surrounded by multiple HP-γ-CD, with hydroxyl oxygens in the “beside” position (green coded) playing a key role in hydrogen bonding with the dye’s oxygen–boron–fluorine moiety. Additionally, the long hydroxypropyl side “chain” (pink coded) aligns parallel to CRANAD-2’s aromatic rings through vdW and hydrophobic interactions, further stabilizing the complex within the 3D host environment.

### Enzyme Assays Using H-GER Complexes

#### α-Amylase Activity

We used the commercially available Phadebas tablet (or blue starch) assay as a gold standard to quantify the fraction of active α-amylase.^58^ As shown in Table S1, the vendor-supplied amylase (380 U/mg) exhibited approximately 83% active enzyme content (320 U/mg), likely due to storage and aging. All amylase concentrations reported in subsequent experiments refer to this active enzyme fraction.

#### Buffer Selection

We used the Phadebas tablet assay to evaluate six media: deionized (DI) water, phosphate-buffered saline (PBS, 10 mM), PBS supplemented with Ca^2+^ (10 mM + 5 mM CaCl_2_), HEPES buffer (50 mM), Tris buffer (50 mM), and imidazole buffer (20 mM). All buffers were adjusted to pH 7.4, except DI water. As shown in Figure 7a, DI water yielded signals 1.1–1.2 times higher than those in buffered media, although the difference was modest. This result is unexpected, as PBS with Ca^2+^ is commonly reported as a preferred buffer for Ca^2+^-dependent α-amylase. We attribute the unexpected results in DI water to two factors: (i) α-amylase has dual activity optima near pH 5 and 7, and pH of DI water ranges from 5.5 to 6.9; and (2) α-amylase exhibits higher activity under low ionic strength conditions.^59,60^ Although signal intensities varied across different media, in one medium the assay consistently yielded a significant color difference between samples with and without amylase (*p* < 0.05).

**Figure 7:**
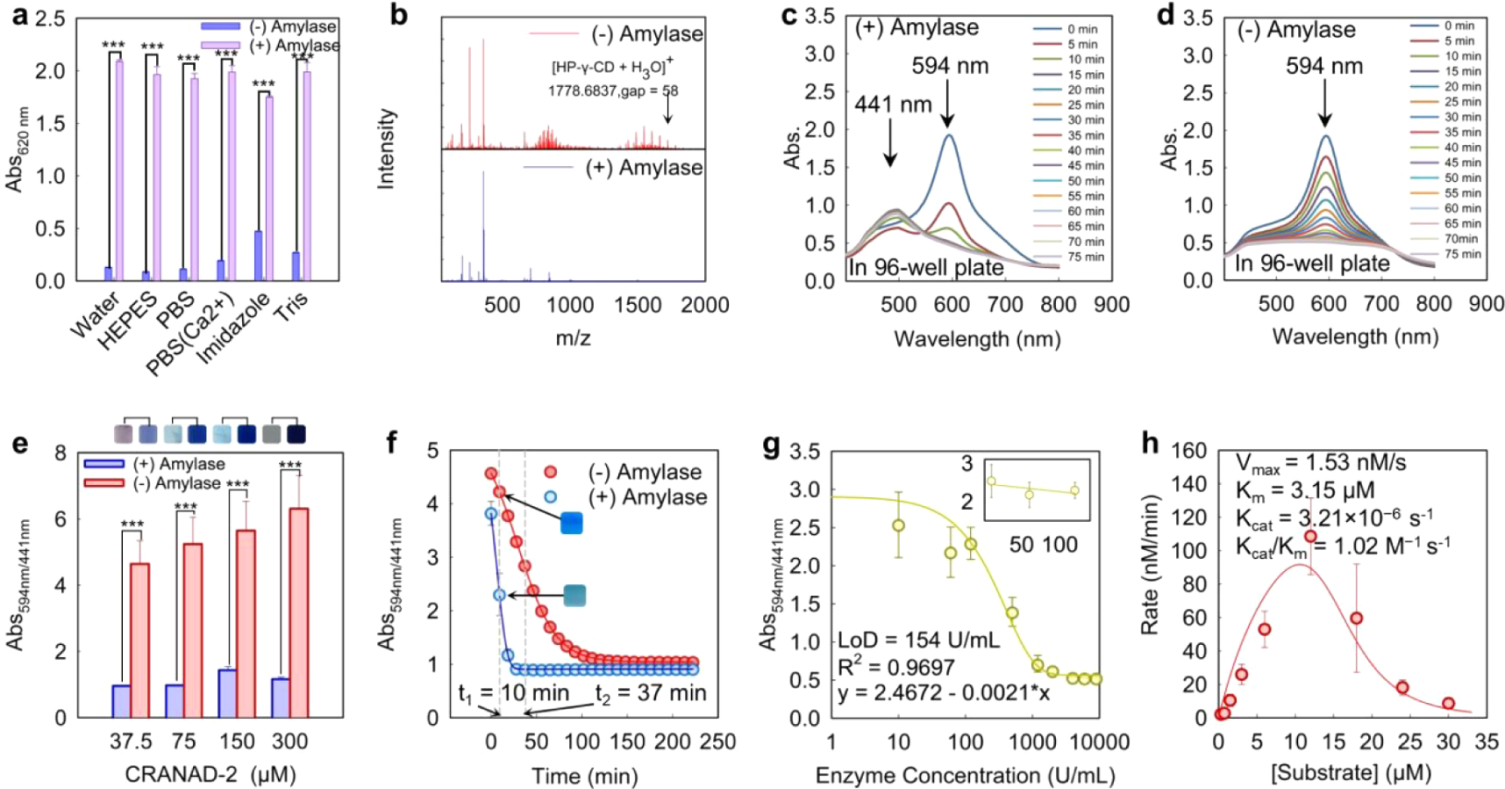
H-GER assays for amylase measurement. (**a**) Buffer optimization for amylase using the standard Phadebas kit. (**b**) ESI-MS spectra of HP-γ-CD before and after α-amylase treatment. The disappearance of the HP-γ-CD-related ion peak at 1778 *m*/*z* and other characteristic peaks indicates enzymatic hydrolysis of the cyclodextrin host. Time-dependent absorbance spectra of CRANAD-2⊂HP-γ-CD complexes (150 μM) in the presence (**c**) and absence (**d**) of α-amylase (415 U/mL). In panel (**c**), enzymatic cleavage induces a decrease in Abs_594 nm_ and the emergence of a new peak at Abs_441 nm._ Panel (**d**) also shows a gradual decrease but without a new peak, due to weak BSA blocking in the 96-well plate. (**e**) The change in Abs_594/441 nm_ and corresponding visible color shift (top) depends on complex concentration. A high ratio reflects more of the initial color, while a low ratio indicates more of the final color. The ratio normalizes concentration effects on colors. A 150 μM dose consistently produced color transformation and was thus defined as the operational concentration for assay performance. (**f**) Time-dependent Abs_594/441 nm_ ratios of CRANAD-2⊂HP-γ-CD complexes (150 μM) with and without α-amylase (415 U/mL). A visible color shift occurred between 10–37 min comparing the two groups, defining the optimal readout window. (**g**) LoD assessment for CRANAD-2⊂HP-γ-CD complexes (150 μM) incubated with increasing α-amylase concentrations. Inset shows a zoomed-in view of the linear range. LoD = 154 U/mL. In panel c-g, we refer to the complex concentration as that of CRANAD-2. (**h**) Determination of *k*_cat_/*K*_m_ = 1.02 M^–1^ s^–1^ for hydrolysis of the CRANAD-2⊂HP-γ-CD complexes by amylase (9960 U/mL) in water at 37 °C. The substrate concentration in panel (**h**) refers to the concentration of HP-γ-CD.

#### Hydrolysis of HP-γ-CD

Hydrolysis of HP-γ-CD by α-amylase was confirmed by electrospray ionization mass spectrometry (MS). As shown in Figure 7b, the control HP-γ-CD sample displayed a characteristic peak at *m*/*z* 1778.6837, attributed to its [M + H_3_O]^+^ ion (calcd *m*/*z* 1779.76) with eight hydroxypropyl substitutions. The observed distribution spacing of ∼58 *m*/*z* corresponds to the mass of a single hydroxypropyl group (−CH_2_CHOHCH_3_), with the central peak assigned to HP-γ-CD bearing four hydroxypropyl units. After incubation with α-amylase at 37 °C for 16 h, these distributed peaks disappeared entirely, indicating complete degradation of the macrocyclic host. No intermediate oligosaccharides such as maltose or maltotriose were detected, likely due to their full hydrolysis to glucose monomer,^61^ which is not readily detectable under the MS conditions used.

#### Operation Concentration

Colorimetric signals of the H-GER assay, based on CRANAD-2⊂HP-γ-CD complexes, were measured by absorbance spectroscopy. As shown in Figure 7c,d, the absorbance profile changes markedly in the presence of amylase. This optical shift parallels the behavior observed with CRANAD-2 complexed with either the cyclic substrate or the linear trisaccharide (Figure 2c). Thus, we will use Abs_594/441 nm_ as a ratiometric indicator of dye aggregation and color change. This colorimetric and optical transition was further supported by transmission electron microscopy (TEM) imaging. Following amylase digestion, the samples showed dense, random aggregates extending to micrometer sizes (Figure S6). In contrast, the predigestion complexes primarily consisted of two populations—subnanometer spheres (0.5–2 nm) and submicrometer cluster-like assemblies—with the latter dominating the measured DLS signal.

We first optimized the operation concentration of CRANAD-2⊂HP-γ-CD complexes for the H-GER assay. Four concentrations (37.5, 75, 150, 300 μM; refer to as CRANAD-2 concentration) were tested in BAS-blocked tubes, and ratiometric absorbance was recorded at equilibrium after amylase addition (9960 U/mL). A minimum concentration of 150 μM complexes was required to consistently produce a strong color change visible to the naked eye within 2 h (Figure 7e). In comparison, lower concentrations (37.5 or 75 μM) failed to yield noticeable or consistent changes, while 300 μM resulted in a dramatic color response but with delayed onset at 4 h. Therefore, 150 μM was selected as the optimal working concentration for all subsequent H-GER experiments.

#### Time Window

We next investigated the optimal time window for readout of color transformation in the H-GER assay. Complexes were incubated with α-amylase (415 U/mL), and UV–vis spectra (350–800 nm) were recorded every 5 min over 4 h at 37 °C. As shown in Figure 7f, the amylase-treated group exhibited a rapid decrease in the Abs_594/441 nm_ ratio, reaching 1 (green color) within 15 min and plateauing by 30 min. In contrast, the control group showed a slower decline, with the ratio remaining around 4 (i.e., blue color) at 15 min. Gradual signal loss observed in the 96-well plate format in 2 h suggested a narrower time window compared to the assay performed in BSA-blocked polypropylene microtubes, likely due to less effective surface passivation on polystyrene. Based on these results, we defined a ratiometric change >1.5 as indicative of a discernible color shift and thus established an optimal detection time window of 10–37 min for naked eyes.

#### Limit of Detection

Using the optimized operation concentration (150 μM) and a 15 min readout time, we evaluated the LoD for amylase by incubating the complexes with varying enzyme concentrations (10–10,000 U/mL) in water at 37 °C in a 96-well plate. As shown in Figure 7g, the ratiometric signal decreased with increasing amylase concentration, and the LoD was determined to be 154 U/mL. We note that this LoD is based on spectroscopic measurements, which is substantially more sensitive than detection by the naked eye. The LoD calculation method is detailed in our previous work and the Supporting Information, Section S6.2.^62,63^ This value is below the oral salivary amylase activity (263–376 U/mL),^64,65^ supporting the clinical relevance of the H-GER system for potential *in vitro* diagnostics in saliva-based forensic applications. However, the current LoD limits its applicability to other biofluids such as urine, blood, and semen, where amylase activity is typically below 0.2 U/mL.^64,65^

#### Enzyme Kinetics

Two key observations prompted us to investigate the kinetics of amylase acting on CRANAD-2⊂HP-γ-CD complexes: (i) a high amylase activity (9960 U/mL) is required to induce a clear visual color change in 15 min, and (ii) the LoD of our system is 3 orders of magnitude higher than that of the commercial Phadebas assay (0.03 U/mL). To understand this discrepancy, we conducted a kinetic analysis using optical data to estimate the amount of cleaved substrate and derive enzymatic parameters. For this, we kept the concentration of CRANAD-2 constant as the optical reporter and varied the amount of HP-γ-CD substrate (0–30 μM) to construct a Michaelis–Menten (MM) curve, using 9960 U/mL of amylase. We assumed that the full range of ratiometric signal change corresponded to complete substrate hydrolysis, allowing proportional estimation of cleaved substrate at any intermediate point. As shown in Figure 7h, the resulting velocity curve deviated from classical MM kinetics and exhibited excess substrate inhibition. The inhibition may result from higher CD-to-dye ratios stabilizing the dye complexes, suppressing ratiometric changes and leading to apparent false-negative results. From the data, we estimated *V*_max_ = 1.53 nM s^–1^, *K*_m_ = 3.15 μM, *k*_cat_ = 3.21 × 10^–6^ s^–1^, and *k*_cat_/*K*_m_ = 1.02 M^–1^ s^–1^ with a known activity of amylase at 320 U/mg. Detailed kinetic experiments are provided in the Supporting Information, Section S6.3. We note that the calculated *k*_cat_/*K*_m_ was 3 orders of magnitude lower than the values reported for amylase digesting cyclodextrins (i.e., 4.2 × 10^3^ M^–1^ s^–1^) by other groups.^66^ This reduced efficiency likely results from the close proximity of CRANAD-2 to the CD substrate, which promotes compact CD clustering and competitive interactions, thereby limiting amylase accessibility and hindering enzyme–substrate binding.^22,58,67^ The slow kinetics are also supported by evidence from DLS measurements and MD simulations. This evidence explains the high enzyme requirement for color change and the comparatively high LoD of our system. Although conjugation techniques used in the standard Phadebas kit minimize interference between the substrate from competitively binding with indicator and enzyme, our H-GER assay offers a key advantage in its simplicity—it requires no complex bioconjugation and serves as a label-free alternative.

### Other Evaluation of H-GER Complexes

#### Assay Specificity

We cross-tested five mammalian proteins—BSA, hemoglobin, β-amylase (cleaves α-1,4-glycosidic bonds from nonreducing ends), trypsin (cleaves C-terminus of Arg/Lys), inactive-α-amylase, and activated Granzyme B (cleaves C-terminus of Asp)—against the H-GER system. As shown in Figure 8a, the positive control—activated α-amylase (9960 U/mL)—induced a strong ratiometric drop from 1.0 to 0.3. In contrast, inactivated α-amylase and Granzyme B produced no optical signal, similar to the negative control (no α-amylase). Although β-amylase, BSA, hemoglobin, and trypsin reduced the ratiometric signal by 0.4, the sample color remained visibly blue (see Figure 8a inset). The red hue in the hemoglobin sample is distinct due to its intrinsic color, not an analytical response. These results demonstrate the high specificity and good compatibility of our complexes with common proteins and off-target enzymes.

**Figure 8:**
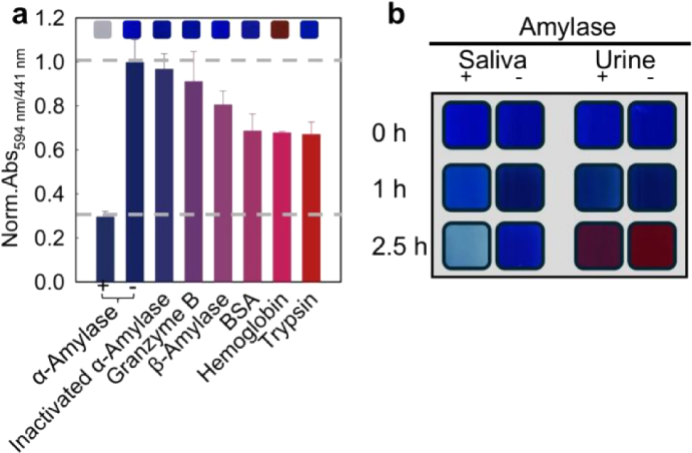
Specificity and matrices effect. (**a**) Specificity analysis of the H-GER assay based on CRANAD-2⊂HP-γ-CD complexes (150 μM) against various enzymes and proteins. Only α-amylase significantly reduced the Abs_594/441 nm_ ratio, while β-amylase (12,000 U/mL), trypsin (1 mg/mL), Granzyme B (1 mg/mL), BSA (1%), hemoglobin (20 mg/mL), and heat-inactivated α-amylase (9960 U/mL) showed minimal to no response. Inset: corresponding color images of the incubated samples. (**b**) Matrix compatibility test in saliva and urine. CRANAD-2⊂HP-γ-CD complexes (150 μM) were incubated with (+) or without (−) α-amylase (9960 U/mL), and color changes were recorded at 0, 1, and 2.5 h. A color change was detected in saliva (pH = 6.7) but not in urine (pH = 8.5). In panel a, b, we refer to the concentration of the CRANAD-2⊂HP-γ-CD complex as that of CRANAD-2.

#### Biological Matrices

To evaluate the clinical applicability of our H-GER system, we conducted preliminary matrix compatibility tests using biological fluids. Specifically, heat-inactivated and 0.2 μm-filtered pooled human saliva (pH = 6.7) and urine (pH = 8.5) were selected due to their physiological relevance to amylase activity. The H-GER assay was performed by incubating 150 μM of CRANAD-2⊂HP-γ-CD complexes with 9960 U/mL of spiked amylase in 400 μL of each medium within BSA-blocked microtubes. White light images were taken at 0, 1, and 2.5 h as the readout. Notably, these fluids also served as reconstitution media for preparing the complexes. As shown in Figure 8b, the system performs well in treated saliva, with the color transitioning from deep blue to light blue/cyan in response to spiked amylase, while samples without amylase stayed deep blue throughout the 2.5 h test. This is significant because saliva is a complex biological matrix containing proteins known to interfere with colorimetric assays through electrostatic and other nonspecific interactions. In contrast, the H-GER complexes were unstable in urine, leading to color shifts in both control and experimental samples. A 25% dilution of urine did not restore stability (Figure S7). To investigate this, we performed pH-dependent stability tests, which showed that the H-GER complexes remain stable within the physiological range of pH 5.0–8.0 (Figure S10). Since the tested urine had a pH of 8.0–8.5, we reasoned that its alkaline nature impairs the stability of the H-GER system. These findings suggest that, while the H-GER assay performs reliably in laboratory buffers and saliva, its performance is reduced in urine fractions, indicating potential need for sample pretreatment such as pH optimization.^68^ Nonetheless, they underscore the assay’s potential for real-world applications, particularly in saliva-based diagnostics, where amylase serves as a clinically relevant biomarker for diabetes, oral health conditions, electronic cigarette use, and forensic saliva testing.

Finally, we would like to discuss the advantages and limitations of our systems and findings. We have validated the H-GER mechanism as an extension of the IDA toolbox for enzyme activity measurement. It functions through enzymatic cleavage of a cyclo-substrate, which releases an aggregachromic reporter into the aqueous phase, leading to a visible color change. This colorimetric platform offers two key advantages: (i) it is simple, label-free, and does not require extensive chemical modification of either the host or the indicator; (ii) it provides high specificity through the combined effects of supramolecular host–guest interactions and enzyme–substrate recognition, enabling reliable and specific performance in aqueous matrices. Compared with common enzyme assays—FRET (costly and dye-dependent), HPLC (labor-intensive and instrument-dependent), and ELISA (measuring concentration, not activity)—H-GER is cost-effective and well-suited for routine use beyond specialized laboratory settings. Building on the insights in this study, we are pursuing several design improvements to enhance the current H-GER system: (i) incorporating aggregachromic dyes with large absorption shifts (>100 nm) to enhance visual contrast; (ii) modifying cyclic host molecules with longer or additional side chains to strengthen interactions with the aggregachromic dye and push the host–colorant ratio toward 1:1; (iii) transitioning from a signal-off to a signal-on strategy using aggregation-induced chromism to reduce the risk of false positives. Our future work will also focus on validating the universality of the H-GER platform by applying this mechanism to additional cyclosubstrates, such as cyclopeptides, cyclooligosaccharides, cyclic nucleotides, and large macrolactones, targeting enzymes including proteases, glycosidases, phosphodiesterases, and esterases. We look forward to reporting on these developments in future studies.

## Conclusion

In summary, we validated the H-GER signal transduction mechanism as an extension of the traditional indicator displacement assay approach for enzyme detection, using α-amylase as a model target. We demonstrated that enzymatic cleavage of CRANAD-2⊂HP-γ-CD complexes triggers a visible color change that correlates with amylase activity. Optimization of the assay involved systematic tuning of multiple parameters, including the synthesis of aggregachromic dye (i.e., CRANAD-2), selection of cyclosubstrates (i.e., HP-γ-CD), solvent conditions (i.e., DMSO), host–guest ratios (i.e., 5:1 by simulations), drying methods (i.e., vacuum centrifuge), and sample stability (i.e., BSA blocking). Structural characterization using ^1^H NMR, DLS, TEM, and MD simulations revealed that CRANAD-2 forms cluster-like complexes with multiple HP-γ-CD molecules, where the HP side chains play a critical role in stabilizing the complex through hydrophobic interactions and hydrogen bonding. No evidence of cavity encapsulation of CRANAD-2 by HP-γ-CD was observed. Despite the distinct and easily detectable color transition, the current LoD of 154 U/mL is approximately 3 orders of magnitude higher than that of the commercial Phadebas blue starch kit (0.3 U/mL). Further kinetic analysis yielded a catalytic efficiency (*k*_cat_/*K*_M_) of 1.02 M^–1^ s^–1^, suggesting that dye–substrate complex formation impedes α-amylase access to the cyclodextrin host, particularly at high complex concentrations above 10 μM. Notably, the H-GER assay demonstrates good specificity, enabled by the combined selectivity of CRANAD-2⊂HP-γ-CD interactions and α-amylase–cyclodextrin recognition. This selectivity allows the system to resist interference from nontarget proteins and function reliably in complex biological media such as saliva. Overall, this work introduces a new colorimetric sensing mechanism and provides foundational insights into the rational design and optimization of H-GER systems for future applications in *in vitro* diagnostics.

## Supplementary Material



## Notes

Materials, H-GER sample preparation, buffer screening, active enzyme determination, LoD calculation, enzyme kinetic assays, synthesis of CRANAD-2, method of sample drying, BSA blocking, solvent effect, optical profiles of other dye⊂CD complexes, optical profiles of tested dyes, chemical structures of dyes, method of MD simulations, TEM, matrix compatibility, urine dilution test, and pH effect on stability (PDF)

## Abbreviations

CRANAD-2: (*T*-4)-[(1*E*,6*E*)-1,7-bis­[4-(dimethylamino)­phenyl]-1,6-heptadiene-3,5-dionato-κO3,κO5]­difluoro-boron
H-GER: host-gated enzymatic release
IDA: indicator displacement assay
CDs: cyclodextrins
HP-γ-CD: hydroxypropyl-γ-cyclodextrin
LT: linear trisaccharide
GM: glucose monomer
RDFs: radial distribution functions
Rg: radius of gyration
SDFs: spatial distribution functions
