# Supplementary material for: Host-Gated Enzymatic Release (H-GER) Enables Colorimetric Transduction for Enzyme Measurement
Source: ACS Appl Mater Interfaces. 2025 Sep 2;17(36):50464–75. doi: 10.1021/acsami.5c12410 (PMC12442016; doi:10.1021/acsami.5c12410)
Supplement: Supplementary file 1 [file am5c12410_si_001.pdf]

Supporting Information

Host-Gated Enzymatic Release (H-GER) Enables Colorimetric Transduction for Enzyme Measurement

Zeyu Zhang,[a,†] Wen Liu,[a,†] Qing Huang,[a] Xiang Zhong,[b] Jin Gu,[c] Ruby Segerman,[a] Jordan Choi,[a] Xing Wang,[a] Zhicheng Jin[a,*]

[a] Department of Chemistry, Georgia State University, Atlanta, Georgia 30303, United States
[b] Department of Chemistry, University of Miami, Coral Gables, Florida 33146, United States
[c] School of Computing, The University of Utah, Salt Lake City, Utah 84112, United States
[†] Authors contributed to the work equally.
[*] Corresponding author's email: zjin4@gsu.edu (Z.J.)

Table of Content

1.Materials 3

2. Synthesis and Characterizations 4

  2.1 CRANAD-2 Synthesis 4

  2.2 Preparation of CRANAD-2⊂HP-γ-CD Complex 4

  2.3 Preparation of other dye⊂CD Complexes 5

  2.4 Dynamic Light Scattering (DLS) Measurement 5

  2.5 Hydrolysis of HP-γ-CD by α-Amylase 6

3. Molecular Dynamic (MD) Simulation 6

4. Buffer Screening 7

5. Amylase Activity Determination 8

6. H-GER-based Enzyme Assays 8

  6.1 Operation concentration determination 8

  6.2 LoD measurement 8

  6.3 Enzyme kinetic assay 9

  6.4 Specificity Tests 10

  6.5 Matrice effect 10

7. References 11

Supporting Tables and Figures 12

Table S1. Calibration of active α-amylase activity using the Phadebas® Amylase Test. 12

Figure S1. Synthetic scheme and ^{1}H NMR spectra of the intermediate and final CRANAD-2 compound. 13

Figure S2. Optimization of sensor preparation and evaluation of BSA blocking effectiveness. 14

Figure S3. Solvatochromic behavior and CD-induced spectral responses of various aggregachromic dyes. 15

Figure S4. Optical properties and molecular structures of four representative dyes. 16

Figure S5. Molecular dynamics (MD) simulation and structural models of the CRANAD-2⊂HP-γ-CD system. 17

Figure S6. TEM imaging. 18

Figure S7. Matrices effect based on a sample dilution. 19

Figure S8. Color stability of the CRANAD-2⊂HP-γ-CD complex in all tested buffers. 20

Figure S9. Poor stability of CRANAD-2⊂HP-γ-CD complexes at a 1:5 dye:CD ratio.......... 21

Figure S10. pH-Dependent stability of the CRANAD-2⊂HP-γ-CD complexes. 22

1.Materials

2,4-Pentanedione (acetylacetone, ≥ 99%, Cat. No. P0052), Nile Red (Cat. No. N0659), Curcumin (Cat. No. C70434), Methylene Blue (Cat. No. A18174.14), dimethyl sulfoxide (DMSO, ≥ 99.7%, Cat. No. BP231-1), and 4-(dimethylamino)benzaldehyde (≥ 98%, Cat. No. A11712.22) were used as chromogenic or solvating agents. Boron trifluoride Ethyl Ether ($BF_3 \cdot Et_2O$, ≥ 98.0%, Cat. No. B0527, TCI) and piperidine (Cat. No. PX1235-4000, Supelco) were employed as catalysts or reactants in condensation reactions. Phosphate-buffered saline (PBS, pH 7.4, Cat. No. 21-040-CV), HEPES buffer (Cat. No. BP310-500, Fisher Bioreagents), imidazole (≥ 98.0%, Cat. No. I0001, TCI), sodium hydroxide (NaOH, 98%, Cat. No. A16037.36, Thermo Scientific), and hydrochloric acid (HCl, 36.5–38.0%, Cat. No. 033257.P6, Thermo Scientific) were utilized in pH adjustment and buffer preparation. Tris(hydroxymethyl)aminomethane (TRIS, 99.97%, Cat. No. 93440-50G, Sigma-Aldrich) and calcium chloride dihydrate ($CaCl_2 \cdot 2H_2O$, Cat. No. BP510-100, Fisher Bioreagents) were used in buffer systems. Organic solvents including acetonitrile (MeCN, ≥ 99.9%, ACS/HPLC grade, Cat. No. AH015-4, Honeywell), dichloromethane (DCM, Certified ACS, stabilized, Cat. No. D37-20, Fisher Chemical), ethyl acetate (EA, Certified ACS, Cat. No. E145-20, Fisher Chemical), hexane (Certified ACS, Cat. No. H292-20, Fisher Chemical), and diethyl ether (Et_2O, anhydrous, ≥ 99.0%, HPLC Grade, Cat. No. E198-4, Fisher Chemical) were used as received. α-Amylase (from Bacillus subtilis, Cat. No. 02100447 380 U/mg), β-amylase (Cat. No. A0448, TCI，380 U/mg), trypsin (Cat. No. 103139, Thermo Fisher), Bovine Serum Albumin (BSA, Cat. No. 05470-5G, Sigma-Aldrich), activated Granzyme B (R&D Systems), hemoglobin (Cat. No. J63838.06, Thermo Scientific), and heat-inactivated α-amylase were used as enzyme analytes. Urine and saliva samples were purchased from Lee BioSolutions, Inc. (Cat. No. 991-03-C and 991-05-F), which supplies 0.2 μm filtered, preservative-free biological fluids from healthy adult donors. All biological samples were handled in accordance with Biosafety Level 2 (BSL-2) guidelines. Sodium sulfate (Na_2SO_4, anhydrous, ≥ 99%, Cat. No. S421-1) was used to dry organic extracts. Deionized water was obtained using a NANO pure Diamond™ purification system (Barnstead, Thermo Scientific). Deuterium oxide (D_2O, ≥ 99% atom D, Cat. No. 16630100) and deuterated dimethyl sulfoxide (DMSO-d_6, ≥ 99% atom D, Cat. No. 2NH0205) were used as NMR solvents. NMR tubes (5 mm, Cat. No. NC0921373, Wilmad Labglass, Vineland, NJ, USA) were used for all NMR measurements. Silica gel for column chromatography (60 Å, 300 mesh) was purchased from Tyger Scientific Inc. (via Amazon, SKU: QY-OS25-50TN). All cyclodextrins (α-CD, β-CD, γ-CD) and their hydroxy propylated derivatives (HP-α-CD, HP-β-CD, HP-γ-CD) were obtained from Millipore Sigma (Burlington, MA, USA). Clear, flat-bottom 96-well microplates (Cat. No. 3596, Corning Costar®, Kennebunk, ME, USA) were

used for absorbance-based assays. All reagents were used as received without further purification unless otherwise noted.

2. Synthesis and Characterizations

2.1 CRANAD-2 Synthesis.

Acetylacetone (10 mmol, 1.03 mL) was dissolved in anhydrous dichloromethane (50 mL) under a nitrogen atmosphere. Boron trifluoride diethyl etherate (15 mmol, 1.85 mL, 1.5 equiv) was added dropwise to the solution over 5 minutes at room temperature. The reaction mixture was then heated to reflux (40 °C) and stirred overnight. Reaction progress was monitored by thin-layer chromatography (TLC) until completion. After cooling to room temperature, the reaction was quenched by the addition of water (15 mL) and stirred for an additional 10 minutes. The organic layer was separated, and the aqueous phase was extracted with dichloromethane (3 × 15 mL). The combined organic layers were washed with water, dried over anhydrous Na_2SO_4, and filtered. The filtrate was concentrated under reduced pressure to afford the crude product, which was used directly in the next step without further purification. ^1H NMR (400 MHz, DMSO-d_6) δ 6.41 (s, 1H), 2.36 (s, 3H), 2.36 (s, 3H).

A drop of piperidine was added to a solution of the 4-dimethylaminobenzaldehyde (4.0 mmol) and 2,2-difluoro-1,3-dioxaboryl-pentadione (0.8 mmol) in dry acetonitrile (10 mL). The reaction mixture was stirred under reflux for 2 hours. The color gradually changed, the solution turned deep blue, similar to the characteristic color of CRANAD-2, indicating completion. The reaction was then quenched with water (50 mL), and the aqueous phase was extracted with dichloromethane (3 × 50 mL). The combined organic layers were dried over anhydrous Na_2SO_4, filtered, and concentrated under reduced pressure. The crude product was purified by silica gel column (5 × 50 cm) chromatography to afford CRANAD-2. ^1H NMR (400 MHz, DMSO-d_6) δ 11.01 (d, J = 15.4 Hz, 2H), 10.86 (d, J = 8.6 Hz, 4H), 9.97 (d, J = 10.6 Hz, 6H), 9.47 (s, 1H), 6.25 (s, 12H).[1]

2.2 Preparation of CRANAD-2⊂HP-γ-CD Complex

A stock solution of CRANAD-2 (3.66 mM) was prepared in DMSO. Notably, the molar extinction coefficient of CRANAD-2 at its absorption maximum is 1.34×10^5 M^{-1} cm^{-1} in DMSO. To make CRANAD-2⊂HP-γ-CD, 16.4 µL of the CRANAD-2 solution (stock sample, 60 nmol) was transferred to a microcentrifuge tube, followed by the addition of 400 µL of HP-γ-CD in DMSO (30 mM, 3 µmol) to achieve a 200:1 molar ratio (HP-γ-CD-to-CRANAD-2). The resulting mixture was sonicated for 40 min and subsequently stirred magnetically at 37 °C overnight in darkness. The solvent was then removed using a vacuum concentrator (Vacufuge, Eppendorf) operated at 30 °C under

aqueous evaporation mode. For subsequent experiments, the dried complex was reconstituted in 400 μL of deionized water or desired media, yielding a deep blue solution. Prior to use, all microcentrifuge tubes or 96-well plates were pre-blocked with 1% (w/v) bovine serum albumin (BSA) solution for 1 hour at room temperature to minimize nonspecific adsorption. After blocking, the blocking solution was removed. Visual colorimetric changes were recorded using an iPhone 14 Pro Max inside a photo box (model: Finnhomy, under consistent lighting conditions), under consistent lighting conditions. Aliquots were also transferred to a 96-well microplate, and absorbance at 594 nm and 441 nm was measured using a plate reader (model Varioskan LUX, Thermo Scientific). Ratiometric values ($Abs_{594/441\ nm}$) were calculated to evaluate the matrix compatibility of the sensing system.

2.3 Preparation of other dye⊂CD Complexes

Complexes of additional dyes with cyclodextrins (i.e., dye⊂CD) were prepared following the same procedure as described in **Section §2.2**. Briefly, each dye stock solution (16.4 μL, 3.66 mM in DMSO, 60 nmol) was then mixed with cyclodextrin solutions in DMSO at varying molar ratios ranging from 1:0.2 to 1:1000 (dye: CD). For each dye—including Nile Red, curcumin, methylene blue, CRANAD-2 (see optical profiles in **Figure S3**)—a total combination of 10 different molar ratios were tested to evaluate the optimal complexation conditions. The mixtures were sonicated for 40 min and stirred overnight at 37 °C in darkness. After complexation, solvents were removed using a vacuum concentrator (Vacufuge, Eppendorf) at 30 °C under aqueous evaporation mode. The resulting dried complexes were reconstituted in 400 μL of DI water or experimental buffer prior to analysis. As the same as with CRANAD-2⊂CD, all microcentrifuge tubes or 96-well plates were pre-blocked with 1% BSA solution for 30 min at room temperature to reduce nonspecific interactions. Aliquots were also transferred to a 96-well microplate, and absorbance at was measured using a plate reader (model Varioskan LUX, Thermo Scientific). Absorbance values were calculated to evaluate the matrix compatibility of the sensing system.

2.4 Dynamic Light Scattering (DLS) Measurement

DLS measurements were performed to evaluate the hydrodynamic diameter (D_H) and polydispersity index (PDI) of both the freshly prepared CRANAD-2⊂HP-γ-CD complexes and free HP-γ-CD in aqueous solution. All measurements were conducted using a dynamic light scattering instrument (model: Litesizer 500, manufacturer: Anton Paar) at a 90°-degree angle, under ambient temperature (25 °C), using un-blocked disposable polystyrene cuvettes (model: 67.754, SARSTEDT AG & Co. KG). For the CRANAD-2⊂HP-γ-CD complex, dried samples prepared at a 200:1 (CD: dye) molar

ratio was reconstituted in 2.5 mL of deionized water. The resulting solution was gently vortexed and sonicated in a water bath (model: 750HT, VWR) for 5 minutes to ensure uniform dispersion. Prior to DLS analysis, the sample was filtered through a 0.2 µm hydrophilic syringe filter (model: SFNY-2013, Cobetter) to remove dust. For the free HP-γ-CD control, 2.5 ml of 30 mM aqueous solution of HP-γ-CD was freshly prepared and filtered through a 0.2 µm syringe filter (model: SFNY-2013, Cobetter) before measurement under identical conditions.

2.5 Hydrolysis of HP-γ-CD by α-Amylase

To assess the enzymatic stability of HP-γ-CD, a hydrolysis test was performed using α-amylase. HP-γ-CD (3.0 µmol, 30 mM in deionized water) was incubated with α-amylase at a final concentration of 9,960 U/mL in a total volume of 400 µL. The mixture was incubated at 37 °C with shaking at 500 rpm for 2.5 h using an Eppendorf ThermoMixer. A control sample containing HP-γ-CD without enzyme was treated under identical conditions. After incubation, samples were directly analyzed by electrospray ionization mass spectrometry in positive mode (ESI^+), using a Waters Micromass Q-TOF micro mass spectrometer.

3. Molecular Dynamic (MD) Simulation

MD simulations were conducted using GROMACS 2024.51 program.[2] Two systems, (1) Bound: CRANAD-2 and HP-γ-CD; (2) Unbound: CRANAD-2 and Linear disaccharide (refer to as CD unit); were prepared in the cubic box by packmol software with the length 16 nm.[3] To ensure consistent initial configurations and minimize artificial clustering, the CRANAD-2 molecules were spatially evenly distributed and positioned in both systems.[4] The OPLS-AA force fields were employed to model bonded and non-bonded interactions.[5] The 1.14*CM1A-LBCC were used to assign atomic partial charges.[6] Non-bonded interactions were excluded in less than three bonds, and standard 1–4 pair interactions were explicitly reintroduced, with LJ interactions scaled by 0.5 and Coulombic interactions scaled by 0.8333 for those pairs, consistent with OPLS-AA parameterization. The Verlet cutoff scheme was employed for neighbor searching, with a 1.6 nm cutoff applied to both van der Waals and electrostatic interactions. All simulations were performed under periodic boundary conditions (PBC) in three dimensions. Long-range electrostatic interactions were calculated using the Particle Mesh Ewald (PME) method, with a fourth-order interpolation scheme, following long-range energy and pressure corrections. LINCS algorithm were applied to constrain hydrogen bonds with an expansion order of four and a maximum constraint angle of 30 degrees to maintain numerical stability during integration.[7] The initial

structures were energy minimized using the steepest descent algorithm with a maximum force convergence threshold 100 kJ/mol/nm. After minimization, the systems were equilibrated in two stages: a) NVT Ensemble (Constant Volume and Temperature): The system was equilibrated for 1 ns at 310.15 K using the velocity-rescale thermostat with a time constant of 5 ps. b) NPT Ensemble (Constant Pressure and Temperature): A subsequent 1 ns equilibration was performed under isotropic pressure coupling at 1 bar using the Parrinello-Rahman barostat with the same thermostat settings as in the NVT stage.[8] Following equilibration, the final production MD simulations were carried out for 200 ns under the same NPT conditions. During this phase, coordinates were saved every 10 ps; Energy terms were recorded every 4 ps; Center-of-mass motion was removed every 2 ps. This protocol enabled robust sampling of dye molecule dynamics while maintaining thermodynamic stability and physical consistency throughout the trajectory.[9, 10]

4. Buffer Screening

All buffer solutions, except deionized water, were adjusted to pH 7.4 using 0.5 M NaOH or 1 M HCl, with pH monitored using a calibrated pH meter (model: Orion Star A211, Thermo Scientific). The buffers tested included PBS, PBS supplemented with 5 mM $CaCl_2$, 50 mM HEPES, 50 mM Tris-HCl, and 50 mM imidazole buffer. Buffers were freshly prepared in deionized water (resistivity ≥ 18.2 MΩ·cm), filtered through a 0.2 μm membrane (model: model: SFNY-2013, Cobetter), and equilibrated to room temperature prior to use.

The α-amylase activity under different buffer conditions was evaluated using the Phadebas® Amylase Test (Magle Life Sciences AB, revised June 2018), following the manufacturer's instructions with modifications for microplate-based absorbance reading. For each condition, 4 mL of buffer solution was added to a 15 mL centrifuge tube, followed by the addition of one Phadebas tablet. Subsequently, 200 μL of α-Amylase solution was added to each tube to achieve a total reaction volume of 4.2 mL. The mixture was vortexed for 10 seconds to initiate tablet dispersion. All tubes were incubated in a well-stirred water bath at 37 °C for exactly 15 minutes. After incubation, the enzymatic reaction was terminated by adding 1.0 mL of 0.5 M sodium hydroxide solution. The tubes were immediately vortexed to ensure uniform mixing and then centrifuged at 1500 g for 5 minutes to sediment insoluble dye fragments. Following centrifugation, 150 μL of the clear supernatant was transferred from each tube into a flat-bottom 96-well plate. The absorbance was measured at 620 nm using a microplate reader (model Varioskan LUX, Thermo Scientific) under ambient conditions. All

measurements were performed in triplicate. The relative α-amylase activity in each buffer condition was calculated based on the net absorbance and normalized to the activity obtained using deionized water as the reference condition.

5. Amylase Activity Determination

The activity of α-amylase was quantified using the Phadebas® Amylase Test (Magle Life Sciences AB), according to the manufacturer's instructions and following the detailed procedure described in **Section §4.0**, Buffer Screening. Briefly, enzyme samples were incubated with Phadebas tablets under controlled conditions, and the reaction was terminated with 0.5 M NaOH. The resulting mixtures were centrifuged to remove insoluble fragments, and the absorbance of the supernatant was measured at 620 nm using a microplate reader (Varioskan LUX, Thermo Scientific). Net absorbance values were obtained by subtracting the blank, and active enzymatic activity (U/mL) was determined by interpolation against a standard curve provided in the instruction or manual of Phadebas® Amylase Test.

6. H-GER-based Enzyme Assays

6.1 Operation concentration determination

For enzyme-responsive colorimetric detection, four concentrations of dried samples (e.g., 37.5, 75, 150, 300 μM of CRANAD-2) prepared at a 200:1 (HP-γ-CD:dye) molar ratio were reconstituted in 400 μL of deionized water and used directly as the reaction medium. The initial blue coloration indicated the successful formation of the CRANAD-2⊂HP-γ-CD complex. α-Amylase powder was precisely weighed and introduced into the solution to achieve a final enzyme concentration of 9,960 U/mL. The volumetric contribution from the enzyme was negligible relative to the total reaction volume (400 μL). Following gentle homogenization, samples were incubated statically at 37 °C. The colorimetric transition was monitored visually over a 3 h period. UV-vis absorption spectra were acquired across the 350-800 nm range using plate reader (Varioskan LUX, Thermo Scientific), with appropriate blanks included for baseline correction.

6.2 LoD measurement

To evaluate the enzyme-responsiveness of the CRANAD-2⊂HP-γ-CD complex, dried samples (150 μM of CRANAD-2)) prepared at a 200:1 molar ratio (CD: dye) were reconstituted in 400 μL of deionized water. Different doses of α-amylase were added

directly during reconstitution to achieve final concentrations of 10, 60, 120, 500, 1200, 2,000, 4,000, 6,000, 9,000, 12,000 U/mL, enabling an enzyme concentration-dependent response study. The resulting solutions were immediately transferred into wells of a 96-well microplate (Cat. No. 3596, Corning Costar®, Kennebunk, ME, USA) pre-blocked with 1% BSA for 1 hour at room temperature. The plate was sealed and placed into plate reader (Varioskan LUX, Thermo Scientific) set to 37 °C. Absorbance spectra were continuously recorded for 16 hours. Each condition was measured in triplicate (n = 3), and absorbance values at 594 nm and 441 nm were extracted for ratiometric analysis ($Abs_{594/441\ nm}$ to monitor enzymatic degradation of the complex over time.

The LoD was determined based on the Clinical and Laboratory Standards Institute (CLSI) EP17-A guideline.[11] First, the limit of blank (LoB) was calculated from replicate measurements of enzyme-free control samples (n = 3), using the formula:

$$LoB = \mu_{blank} + 1.645 \times \sigma_{blank}$$

where μ_{blank} and σ_{blank} represent the mean and standard deviation of the blank group, respectively.

Subsequently, 3 data points corresponding to low α-amylase concentrations (10, 60, 120 U/mL) were selected for linear regression analysis. Absorbance measurements were taken at 20 mins; a time point within the experimentally defined operational window (10-37 mins). The resulting calibration curve followed a linear model of the form:

$$A = a + b \times [Amylase],$$

where a and b are constant derived from the fit. The LoD was obtained by substituting the previously calculated LoB signal value into this regression equation and solving for the corresponding enzyme concentration.

6.3 Enzyme kinetic assay

To evaluate the enzymatic kinetics of the CRANAD-2⊂HP-γ-CD system, a series of reactions were performed using increasing substrate concentrations under a fixed enzyme level (9,960 U/mL). The CRANAD-2⊂HP-γ-CD complexes were weighed and reconstituted in deionized water to obtain final substrate concentrations ranging from 1.5, 3, 6, 12, 18, 24, 30 μM (refer to as CRANAD-2 concentration). α-Amylase powder was added to each sample to reach a constant final enzyme concentration of 9,960 U/mL. The total reaction volume for each condition was 400 μL.

Following gentle vortexing to ensure complete dispersion, each sample was transferred into a 96-well flat-bottom microplate (Corning Costar, Cat. No. 3596) pre-blocked with

1% BSA for 1 hour at room temperature. The microplate was sealed and incubated at 37 °C inside a plate reader (Varioskan LUX, Thermo Scientific). Absorbance spectra were continuously recorded for 16 hours within the 350–800 nm range.

Ratiometric analysis ($Abs_{594/441\ nm}$) was used as the optical readout for substrate degradation. Reaction velocities were calculated based on the initial rate of signal change within the experimentally defined linear window. All measurements were performed in triplicate (n = 3), and resulting data were used to construct a Michaelis–Menten plot and fit for kinetic parameters (e.g., $k_{cat} = V_{max}/[E]$ with α-amylase to be 320 U/mg and 55 kDa; K_m is defined as the Michaelis constant, which represents the substrate concentration at which the enzyme reaction rate is half of its maximum velocity, V_{max}).[12]

6.4 Specificity Tests

To evaluate the specificity of the CRANAD-2⊂HP-γ-CD colorimetric sensing system, the response of the complex to various non-target proteins was tested under identical conditions. Dried samples (150 µM of CRANAD-2) were prepared at a 200:1 molar ratio (CD: dye) were first reconstituted in 400 µL of deionized water, after which BSA, inactive α-amylase, β-amylase, trypsin, activated Granzyme B, and hemoglobin were individually added to achieve a final protein concentration of 100 µM, simulating non-target protein interference conditions.. The reconstituted solutions were transferred into 2 mL microcentrifuge tubes (Eppendorf Safe-Lock, Cat. No. 0030120094) that had been pre-blocked with 1% BSA for 1 hour to prevent nonspecific adsorption.

The tubes were incubated at 37 °C with agitation at 500 rpm using an Eppendorf ThermoMixer (model: F2.0) for 2.5 h. A positive control group containing α-amylase at an active concentration (9,960 U/mL) was included to validate the sensor's responsiveness. A negative control group consisting of complex samples reconstituted in water without any added protein was also prepared to establish the baseline signal. At endpoint, aliquots were transferred to a 96-well microplate for absorbance measurement. The absorbance at 594 nm and 441 nm was recorded using a plate reader (model: Varioskan LUX, Thermo Scientific), and ratiometric values ($Abs_{594/441\ nm}$) were calculated to assess signal response. In parallel, color changes were documented visually using an iPhone 14 Pro Max under standardized lighting conditions within a custom-built photo box (model: Finnhomy).

6.5 Matrice effect

To assess the applicability of the CRANAD-2⊂HP-γ-CD colorimetric sensor in biologically relevant matrices, human saliva and urine were tested as media using a

heat-deactivated enzyme-response H-GER assay. Dried samples (150 μM of CRANAD-2) prepared at a 200:1 molar ratio (CD: dye) were reconstituted in 400 μL of heat-inactivated biological fluids (i.e., saliva or urine), with α-amylase added to adjust the final enzyme concentration to 9,960 U/mL. Note that pooled saliva and urine media were first filtered through a 0.2 μm syringe filter (model: SFNY-2013, Cobetter) to remove debris and then subjected to thermal treatment at 95 °C for overnight to inactivate endogenous enzymes. The resulting solutions were transferred into 2 mL BSA-blocked microcentrifuge tubes (model: Eppendorf Safe-Lock, Cat. No. 0030120094) and incubated at 37 °C and 500 rpm using a Thermomixer (Eppendorf, model: F2.0) for 2.5 h. Visual colorimetric changes were recorded using an iPhone 14 Pro Max inside a photo box (model: Finnhomy), under consistent lighting conditions.

7. References

(1) Ran, C.; Xu, X.; Raymond, S. B.; Ferrara, B. J.; Neal, K.; Bacskai, B. J.; Medarova, Z.; Moore, A. Design, Synthesis, and Testing of Difluoroboron-Derivatized Curcumins as Near-Infrared Probes for in Vivo Detection of Amyloid-β Deposits. *JACS* **2009**, *131* (42), 15257-15261.

(2) Páll, S.; Zhmurov, A.; Bauer, P.; Abraham, M.; Lundborg, M.; Gray, A.; Hess, B.; Lindahl, E. Heterogeneous parallelization and acceleration of molecular dynamics simulations in GROMACS. *J. Chem. Phys.* **2020**, *153* (13).

(3) Martínez, L.; Andrade, R.; Birgin, E. G.; Martínez, J. M. PACKMOL: A package for building initial configurations for molecular dynamics simulations. *J. Comput. Chem.* **2009**, *30* (13), 2157-2164.

(4) Abascal, J. L. F.; Vega, C. A general purpose model for the condensed phases of water: TIP4P/2005. *J. Chem. Phys.* **2005**, *123* (23).

(5) Jorgensen, W. L.; Maxwell, D. S.; Tirado-Rives, J. Development and Testing of the OPLS All-Atom Force Field on Conformational Energetics and Properties of Organic Liquids. *JACS* **1996**, *118* (45), 11225-11236.

(6) Dodda, L. S.; Vilseck, J. Z.; Tirado-Rives, J.; Jorgensen, W. L. 1.14*CM1A-LBCC: Localized Bond-Charge Corrected CM1A Charges for Condensed-Phase Simulations. *J. Phys. Chem. B.* **2017**, *121* (15), 3864-3870.

(7) Hess, B.; Bekker, H.; Berendsen, H. J. C.; Fraaije, J. G. E. M. LINCS: A linear constraint solver for molecular simulations. *J. Comput. Chem.* **1997**, *18* (12), 1463-1472.

(8) Humphrey, W.; Dalke, A.; Schulten, K. VMD: Visual molecular dynamics. *J. Mol. Graph.* **1996**, *14* (1), 33-38.

(9) Gowers, R. J.; Linke, M.; Barnoud, J.; Reddy, T. J. E.; Melo, M. N.; Seyler, S. L.; Domański, J.; Dotson, D. L.; Buchoux, S.; Kenney, I. M.; Beckstein, O. MDAnalysis: A Python Package for the Rapid Analysis of Molecular Dynamics Simulations. In *Proc. 15th Python Sci. Conf.*, 2016.

(10) Brehm, M.; Thomas, M.; Gehrke, S.; Kirchner, B. TRAVIS—A free analyzer for

trajectories from molecular simulation. *J. Chem. Phys.* **2020**, *152* (16).
(11) Moretti, M.; Sisti, D.; Rocchi, M. B.; Delprete, E. CLSI EP17-A protocol: A useful tool for better understanding the low end performance of total prostate-specific antigen assays. *Clin Chim Acta.* **2011**, *412* (11), 1143-1145.
(12) Koerber, S. C.; Fink, A. L. The analysis of enzyme progress curves by numerical differentiation, including competitive product inhibition and enzyme reactivation. *Anal. Biochem.* **1987**, *165* (1), 75-87.

Supporting Tables and Figures

Table S1. Calibration of active α-amylase activity using the Phadebas® Amylase Test.

Apparent Enzyme Concentration (U/L)	$Abs_{620\,nm}$	Active enzyme concentration (U/L)
0	0	0
30	0.2822	24.9±0.8
60	0.5822	50.8±1.0
120	1.1959	103.3±3.4

Note: Apparent enzyme concentrations with the corresponding absorbance values at 620 nm. The actual active enzyme concentrations were interpolated from the standard curve provided by the test kit instruction. Data are presented as mean ± standard deviation (n = 3).

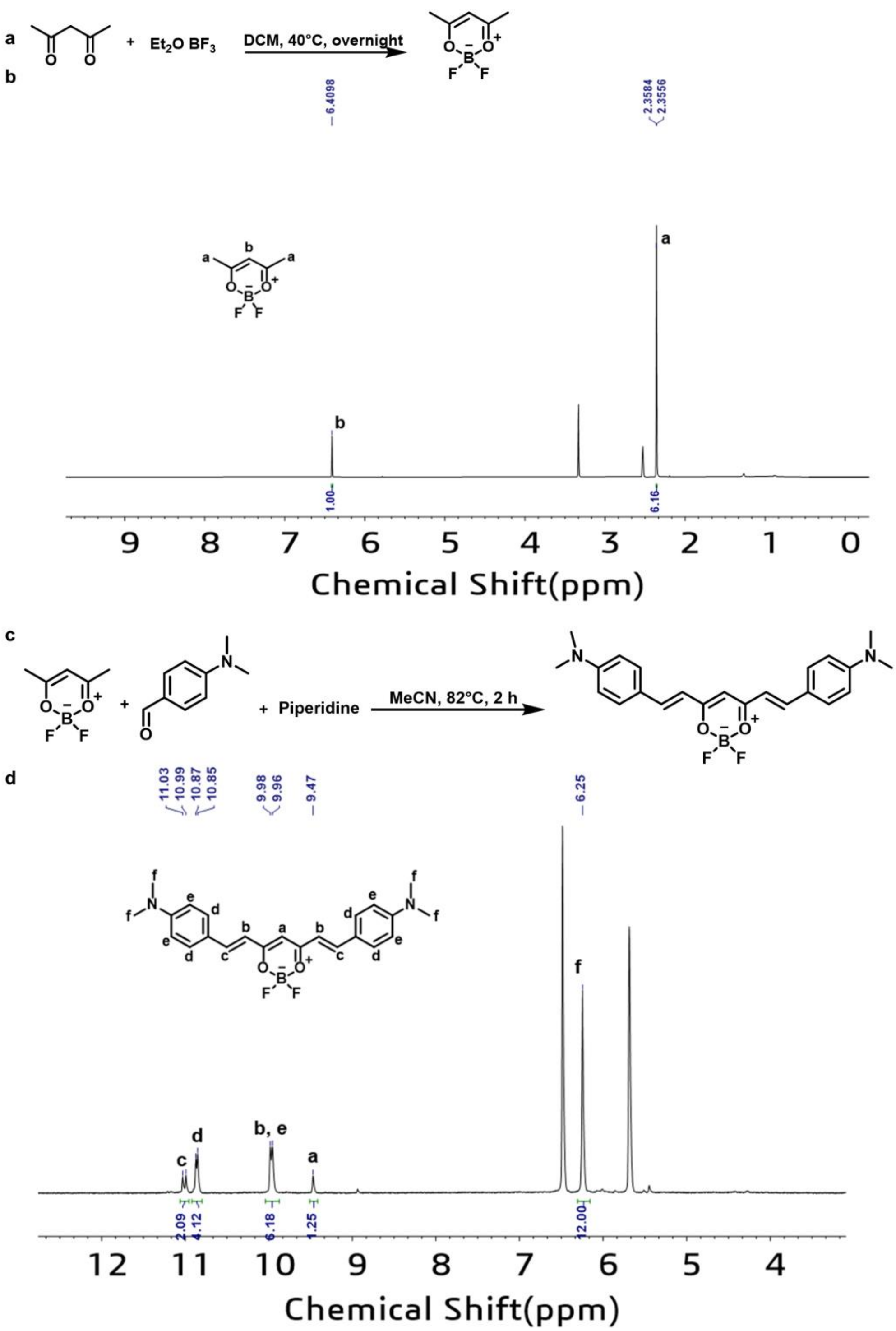

Figure S1. Synthetic scheme and ^{1}H NMR spectra of the intermediate and final CRANAD-2 compound. **(a)** Synthesis of difluoroboron β-diketonate intermediate by reacting acetylacetone with boron trifluoride diethyl etherate in DCM at 40 °C overnight. **(b)** ^{1}H NMR spectrum of the difluoroboron β-diketonate intermediate in DMSO-d_6. **(c)** Final condensation reaction between the boron-complexed diketone and 4-(dimethylamino)benzaldehyde in the presence of piperidine as base, in MeCN at 82 °C for 2 h. **(d)** ^{1}H NMR spectrum of the final product CRANAD-2 in DMSO-d_6.

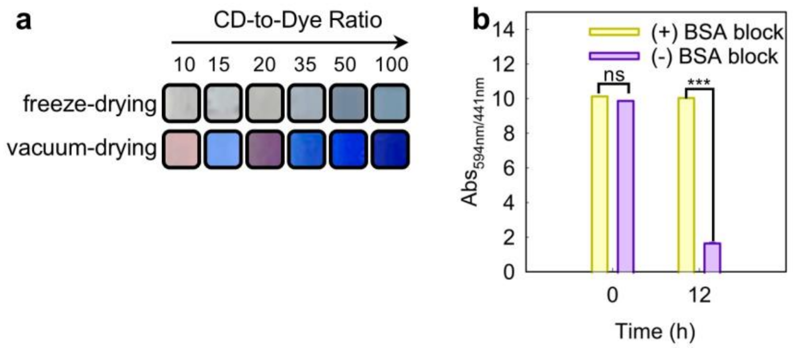

Figure S2. Optimization of sensor preparation and evaluation of BSA blocking effectiveness. (a) Visual comparison of CRANAD-2⊂HP-γ-CD complex samples prepared by freeze-drying versus vacuum-drying at various CD-to-dye molar ratios (10:1 to 100:1). Color intensity increases with higher CD ratios, with vacuum-drying samples showing a fully restored blue coloration. **(b)** Effect of BSA surface blocking on H-GER stability over 12 hours. Ratiometric absorbance ($Abs_{594/441\ nm}$) was measured at 0 and 12 h for sensors stored in BSA-blocked (+) and unblocked (–) polypropene microtubes. Blocking with BSA significantly reduced signal loss over time and thus improved colloidal stability.

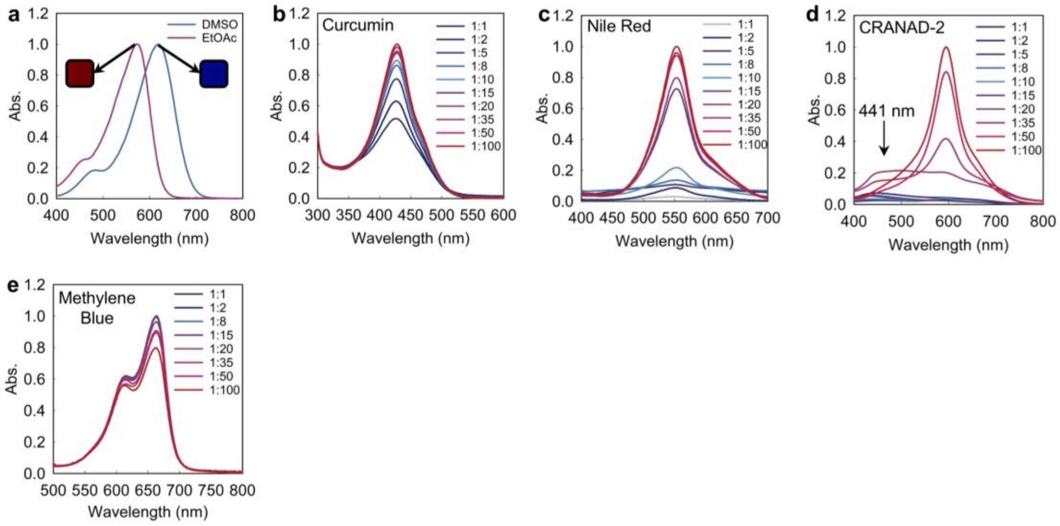

Figure S3. Solvatochromic behavior and CD-induced spectral responses of various aggregachromic dyes. (a) Absorption spectra of CRANAD-2 in two solvents with distinct polarity (DMSO vs EtOAc), showing significant solvatochromic shift. Inset depicts corresponding solution colors. Optical profiles of four dye molecules—**(b)** Curcumin, **(c)** Nile Red, **(d)** CRANAD-2, and **(e)** Methylene Blue—measured in the presence of increasing concentrations of HP-γ-CD (CD: dye molar ratio ranges from 1 to 100). Among them, only CRANAD-2 exhibited an emerging peak at 441 nm with increased intensity at low CD concentrations. This caused a progressive blue shift of the main peak as the CD:dye ratio decreases and thus a color transformation. In contrast, Curcumin and Nile Red showed only intensity changes without peak shifts, while Methylene Blue remained spectrally unchanged because its water soluble.

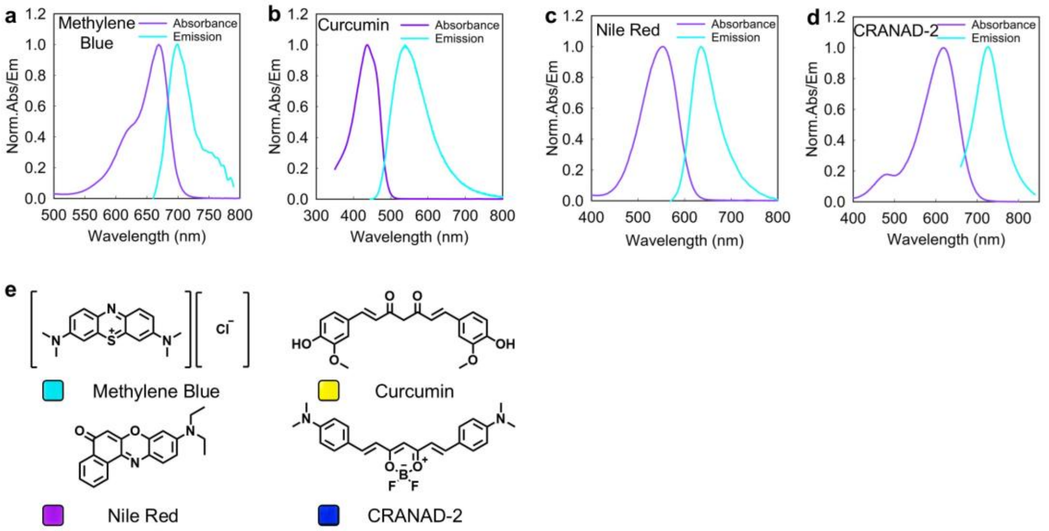

Figure S4. Optical properties and molecular structures of four representative dyes. (a–d) Normalized absorbance (purple) and fluorescence emission (cyan) spectra of **(a)** Methylene Blue, **(b)** Curcumin, **(c)** Nile Red, and **(d)** CRANAD-2 in DMSO. **(e)** Chemical structures and corresponding experimental solution colors of the dyes: Methylene Blue (cyan to blue), Curcumin (yellow), Nile Red (magenta), and CRANAD-2 (blue).

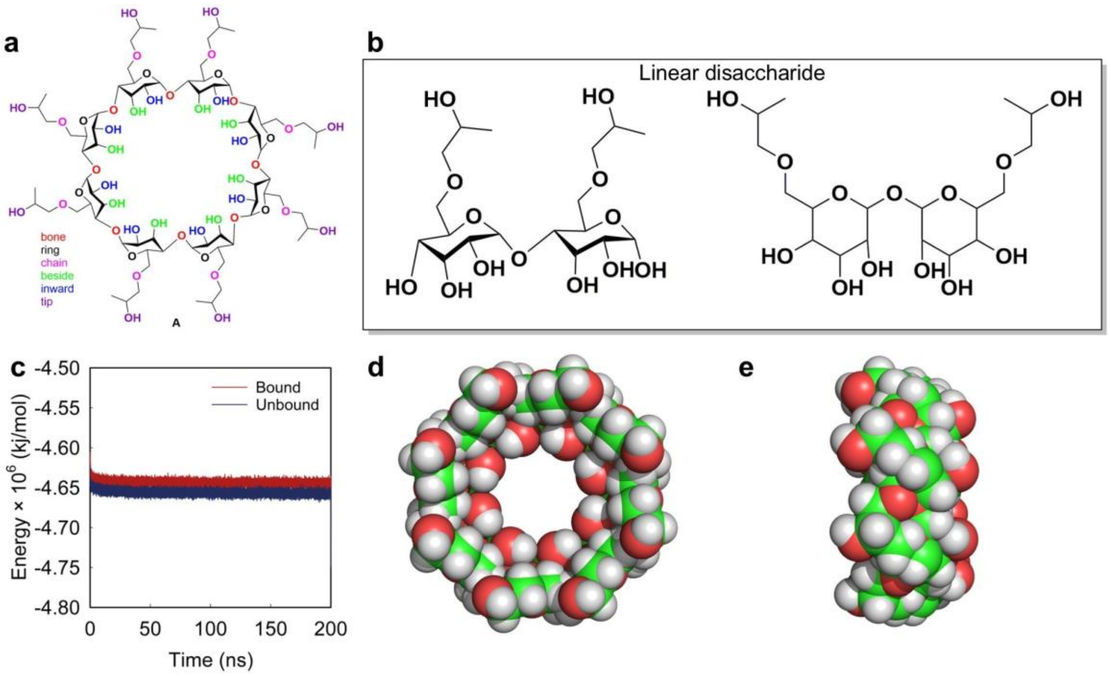

Figure S5. Molecular dynamics (MD) simulation and structural models of the CRANAD-2⊂HP-γ-CD system. **(a)** Structural model of HP-*γ*-CD with different types of oxygen atoms in the designated positions with coded colors. **(b)** Chemical structures of linear disaccharides used in unbound systems, which simulate an amylase-cleaved HP-*γ*-CD molecules lacking macrocyclic encapsulation capability. **(c)** Potential energy profiles of two simulated systems over 200 ns: the bound system consisting of 200 HP-*γ*-CD molecules and 10 CRANAD-2 molecules, and the unbound system composed of 800 linear disaccharide (or CD unit) molecules and 10 CRANAD-2 molecules. **(d, e)** Initial 3D structures of HP-*γ*-CD from the front view **(d)** and side view **(e)**, highlighting its toroidal geometry and internal cavity features.

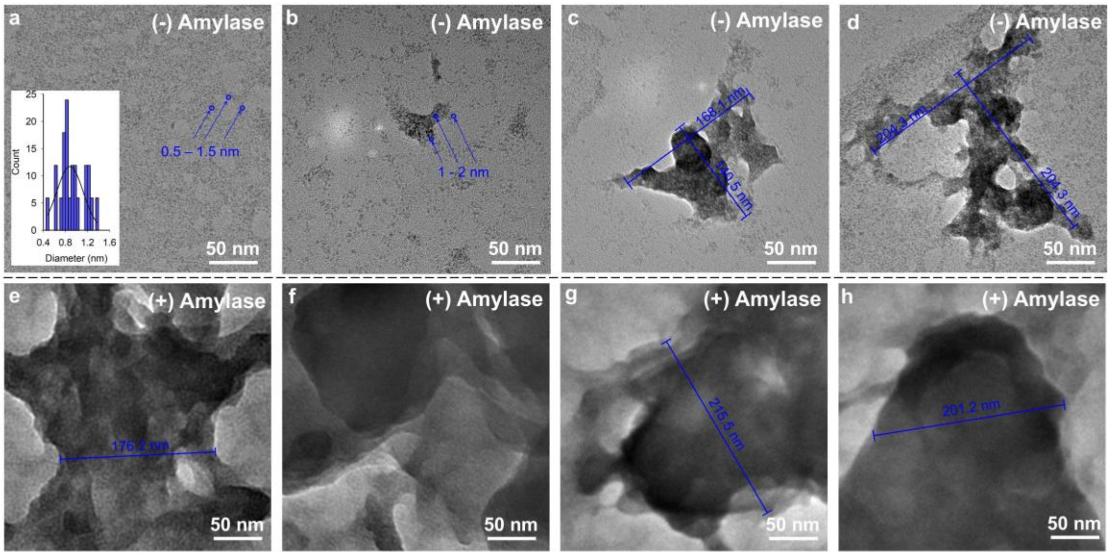

Figure S6. TEM imaging. TEM imaging of CRANAD-2⊂HP-γ-CD complexes before (**a–d**) and after (**e–h**) α-amylase digestion. Prior to enzyme treatment, the complexes exhibited two main populations: sub-nanometer spherical species (0.5–2 nm; **a,b**) and sub-micrometer cluster-like assemblies (150–250 nm; **c,d**). Following α-amylase digestion, the complexes formed dense, irregular aggregates extending to micrometer scales, consistent with CRANAD-2 release and subsequent aggregation in aqueous media, which accounts for the observed color shift.

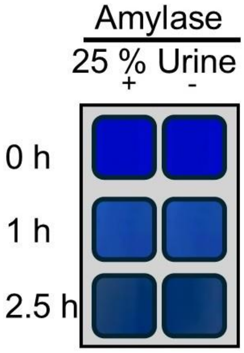

Figure S7. Matrices effect based on a sample dilution. (a) Matrix compatibility test in 25 % dilution of urine. CRANAD-2⊂HP-γ-CD complexes were incubated with or without α-amylase (9,960 U/mL), and color changes were recorded at 0, 1, and 2.5 h. No discernible color change was observed in 25% diluted urine with and without amylase.

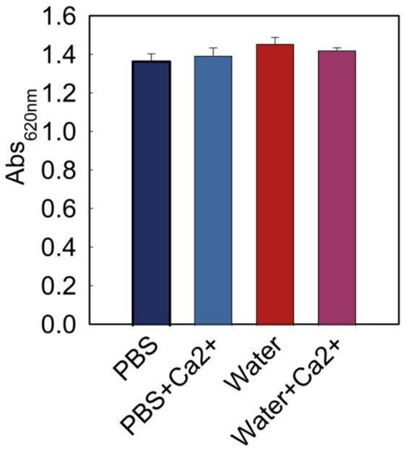

Figure S8. Color stability of the CRANAD-2⊂HP-γ-CD complex in all tested buffers. Absorbance at 620 nm was measured for complexes prepared in PBS, PBS supplemented with Ca^{2+}, deionized water, and water supplemented with Ca^{2+} in 15 min. The results show comparable absorbance values across all tested conditions, indicating that neither buffer type nor the presence of Ca^{2+} significantly influenced the optical stability of the sensing system.

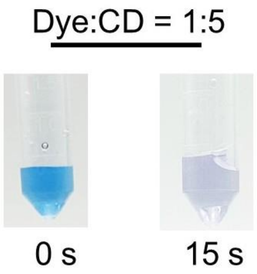

Figure S9. Poor stability of CRANAD-2⊂HP-γ-CD complexes at a 1:5 dye:CD ratio. (a) Upon dissolution, the 1:5 CRANAD-2⊂HP-γ-CD complexes immediately exhibit a transient light-blue color (0 s), which rapidly fades to gray within 15 s. This observation indicates that the 1:5 ratio produces unstable complexes with limited color persistence.

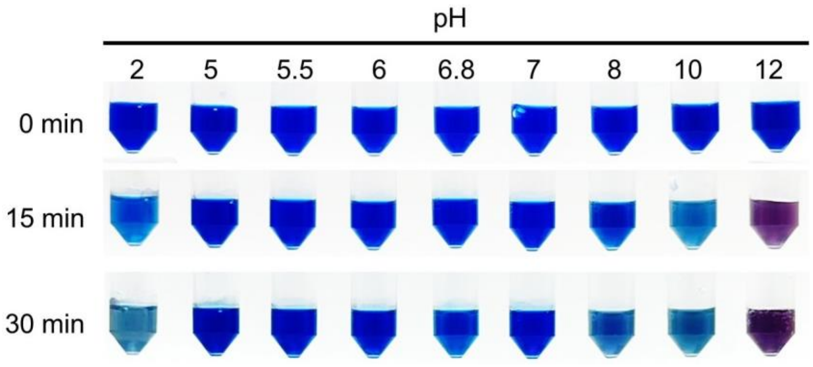

Figure S10. pH-Dependent stability of the CRANAD-2⊂HP-γ-CD complexes. (a) Photographs of CRANAD-2⊂HP-γ-CD complexes incubated in buffers of varying pH (2–12) for 0, 15, and 30 min. The complexes remained stable and retained their blue coloration in the near-neutral range (pH 5.0–8.0).
